# Mendelian Randomization Analysis Provides Insights into the Pathogenesis of Serum Levels of Branched-Chain Amino Acids in Cardiovascular Disease

**DOI:** 10.3390/metabo13030403

**Published:** 2023-03-09

**Authors:** Wenxi Jiang, Ke Lu, Zhenhuang Zhuang, Xue Wang, Xun Tang, Tao Huang, Pei Gao, Yuan Wang, Jie Du

**Affiliations:** 1 Key Laboratory of Remodeling-Related Cardio-Vascular Diseases, Ministry of Education, Beijing Collaborative Innovation Centre for Cardiovascular Disorders, Beijing Anzhen Hospital, Capital Medical University, No. 2 Anzhen Road, Chaoyang District, Beijing 100029, China; 2 Beijing Institute of Heart, Lung and Blood Vessel Disease, No. 2 Anzhen Road, Chaoyang District, Beijing 100029, China; 3 Department of Epidemiology and Biostatistics, School of Public Health, Peking University, No. 38 Xueyuan Road, Haidian District, Beijing 100083, China; 4 Center for Real-World Evidence Evaluation, Peking University Clinical Research Institute, Peking University Health Science Center, Peking University, Beijing 100083, China

**Keywords:** cardiovascular disease, branched-chain amino acid, mendelian randomization, single-nucleotide polymorphism, neuroendocrine

## Abstract

Several observational studies have indicated an association between high serum levels of branched-chain amino acids (BCAAs) and an increased risk of cardiovascular disease (CVD). To assess whether theses associations reflect causality, we carried out two-sample Mendelian randomization (MR). Single-nucleotide polymorphisms (SNPs) associated with BCAA were evaluated in 10 studies, including 24,925 participants. The association between SNPs and coronary artery disease (CAD) were assessed using summary estimates from the CARDIoGRAMplusC4D consortium. Further MR analysis of BCAAs and seven CVD outcomes was performed. The BCAA-raising gene functions were also analyzed. MR analyses revealed a risk-increasing causal relationship between serum BCAA concentrations and CAD (odds ratio 1.08; 95% confidence interval (CI) 1.02–1.14), which was partly mediated by blood pressure and type 2 diabetes. BCAA also demonstrated a causal relationship with ischemic CVD events induced by plaque rupture and thrombosis (false discovery rate <0.05). Two BCAA-raising genes (*MRL33* and *CBLN1*) were preferentially associated with myocardial infarction risk in the presence of atherosclerosis (*p* < 0.003). Functional analysis of the BCAA-raising genes suggested the causal involvement of two pathophysiological pathways, including glucose metabolism (*PPM1K* and *TRMT61A*) related to plaque progression, and the newly discovered neuroendocrine disorders regulating blood pressure (*MRPL33*, *CBLN1*, and *C2orf16*) related to plaque rupture and thrombosis. This comprehensive MR analysis provided insights into the potential causal mechanisms linking BCAA with CVD risk and suggested targeting neuroendocrine disorders as a potential strategy for the prevention of CVD. These results warrant further studies to elucidate the mechanisms underlying these reported causal associations.

## 1. Introduction

Cardiovascular disease (CVD) is the leading cause of morbidity and mortality worldwide [1]. Data from cohort and case–control studies have suggested that raised circulating concentrations of branched-chain amino acids (BCAAs) are associated with an increased risk of coronary artery disease (CAD) [2,3,4,5]. As important energy metabolism substrates, BCAAs, including leucine, isoleucine, and valine, are important regulators of systemic metabolism, energy expenditure, and muscle protein synthesis. Abnormal accumulation of BCAAs increases the risk of CAD and subsequent serious cardiovascular events, such as myocardial infraction (MI), sudden death, or stroke, due to the rupture of atherosclerotic plaques and thrombus formation [5,6]. BCAA catabolism has been shown to damage cardiovascular endothelial cells in mice and promotes the development of CVD [7]. However, data from experimental models cannot explain the causal relationship between circulating BCAAs and CVD in a heterogeneous population in a complex environment.

Well-powered, genome-wide association studies (GWAS) have identified hundreds of single-nucleotide polymorphisms (SNPs) associated with CVD-related traits or circulating BCAAs [8,9]. This creates the opportunity to test potentially causal genetic relationships between these SNPs and other clinically relevant cardiovascular traits using a Mendelian randomization (MR) approach. Multiple serum BCAA-associated genetic variants can thus be used as instrumental variables to assess the relationship between serum BCAA concentrations and CVD risk. Recent studies have focused on how BCAAs promote insulin resistance and diabetes, which in turn cause CVD [10]. However, the molecular mechanism underpinning this causal association remains unclear, and there is thus a need to explore and understand this mechanism to identify potential new interventions for patients with CVD. Based on the results of projects such as the Gene-Tissue Expression Project (GTEx) [11], the Human Protein Atlas (HPA) [12], and Phenome Wide Association Studies (PWAS), we hypothesized that this would provide an opportunity to explore the pathophysiological mechanisms by which BCAA affects CVD.

In the present study, we carried out an MR analysis using data generated from various GWAS datasets to detect potentially causal links between BCAAs and CAD. We further aimed to identify intermediate phenotypes that may mediate any causal effects of BCAAs on CAD. We also investigated the potential causal associations between BCAAs and a broad range of CAD-related clinical events, including ischemic events (MI, heart attack, stroke, deep venous thrombosis, or pulmonary embolism) and bleeding events (intracerebral and subarachnoid hemorrhages). We then evaluated the relationship between BCAA-raising SNPs and the risk of MI (GWAS data with MI vs. CAD only) due to plaque rupture and thrombosis. In the present study, we sought to address the hypothesis that genetically determined BCAAs may lead to CAD and subsequent ischemic events through multiple pathophysiological pathways to vulnerable plaques and thrombus formation. Therefore, we seek to BCAA-raising locus to putative effector genes through integrated analysis of expression data from disease-relevant tissues in order to broaden our understanding of the pathophysiological basis of the impact of BCAAs on the biological mechanisms responsible for plaque initiation, progression, rupture, and thrombosis in patients with CVD.

## 2. Methods

### 2.1. Study Overview

**Figure 1:**
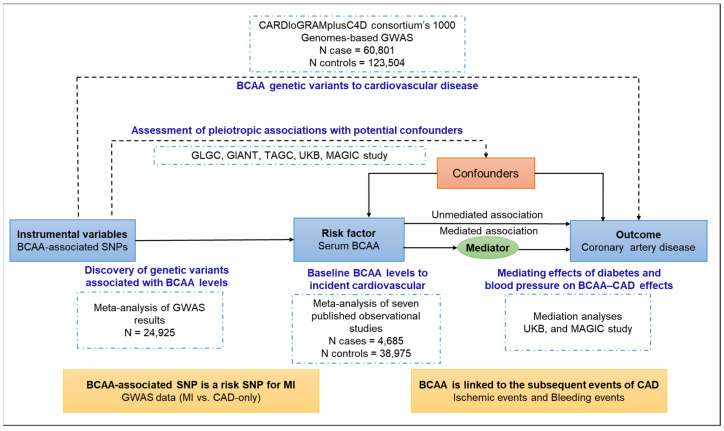
Study design. Genetic variants are assigned at birth, are largely randomly assorted in a population, and can be used as instrumental variables to estimate the causal association of an exposure (e.g., branched-chain amino acids, BCAAs) with an outcome of interest (e.g., coronary artery disease, CAD). This approach rests on three assumptions: the genetic variants must be associated with the exposure, must not be associated with confounders, and must influence the risk of the outcome through the exposure and not through other pathways. Mendelian randomization can be extended to estimate the association between exposure and outcome via a given mediator (e.g., blood pressure and type 2 diabetes). Furthermore, the causal link between BCAAs and CAD-related clinical events was evaluated, and the association between BCAA-raising single-nucleotide polymorphisms (SNPs) and the risk of myocardial infarction (MI) caused by plaque rupture/thrombosis was investigated.

We derived causal estimates for the association of serum BCAAs with the risk of CAD using six different MR approaches based on multiple serum BCAA-associated genetic variants, identified intermediate phenotypes that may mediate causal effects, and compared the association with the results of a meta-analysis of observational studies. We further evaluated the causal link between BCAAs and CAD-related clinical events and the association between BCAA-raising SNPs and the risk of MI caused by plaque rupture/thrombosis (Figure 1). Finally, we carried out a functional analysis of effector genes using tissue-specific transcriptome, proteome, and phenotype-related GWAS datasets. We used summary estimates for the effects of genotype on exposures and outcomes from the largest available meta-analyses of previous GWAS studies, focusing on datasets including participants predominantly of European descent, to ensure consistent allele frequencies across the datasets and avoid possible modifications of genetic effects by ancestral origin.

### 2.2. GWAS Summary Level Data on BCAA and CAD

The current analysis involved the use of publicly available, de-identified data (Table 1). All the original studies included appropriate ethical review and informed consent. The serum BCAA-associated SNP set was derived from a previous GWAS [8]. The primary outcome data (CAD) for the analysis were retrieved from the CARDIoGRAMplusC4D Consortium database. The analytic sample included up to 60,801 cases of CAD (approximately 70% with MI) and 123,504 non-cases from 48 cohort and case–control studies. Most participants were of European (77%), South Asian (13%), or East Asian (6%) ancestry [9]. Table 1 provides further details on the sources of the GWAS summary statistics.

### 2.3. GWAS Summary Level Data on CAD Clinical Event and Mediation

CAD clinical outcome factors consisted of seven diseases that have been published in relevant GWAS summary data. Stroke was obtained from the MEGASTROKE consortium [13]. Intracranial haemorrhage and cardiac arrest were obtained from the FinnGen consortium (https://finngen.gitbook.io/documentation/, accessed on 26 May 2022). Atrial fibrillation was obtained from the GWAS Catalog (GCST006414) [14]. Heart attack/myocardial infarction, deep venous thrombosis, and pulmonary embolism were obtained from the UK Biobank consortium (http://www.nealelab.is/uk-biobank, accessed on 26 May 2022) (Table 1).

Potential mediator GWAS summary statistics data were collected from the following resources: the Giant Investigation of Anthropometric Traits (GIANT) consortium [15] (Body mass index, waist-to-hip ratio), the Meta-Analyses of Glucose and Insulin-related traits Consortium [16] (MAGIC Fasting glucose Fasting insulin HbA1C 2h-glucose HOMA-IR), the UK Biobank consortium (Blood pressure), the Tobacco and Genetics Consortium (TAGC, smoking) [17], and the Global Lipids Genetics Consortium (GLGC LDL, HDL, triglycerides, Total cholesterol) [18].

**Table 1:** Details of studies and datasets used for analyses.

Exposure/Outcome	Consortium	Participants	Web Source If Publicly Available
**Mendelian randomization analysis (BCAA to CAD)**			
Serum BCAA	MAGNETIC NMR-GWAS [8]	24,925 individuals of European ancestry	http://www.computationalmedicine.fi/, accessed on 27 August 2021
Coronary artery disease	CARDIoGRAMplusC4Dconsortium’s 1000 Genomes-based GWAS [9]	184,305 individuals (60,801 CAD cases and 123,504 non-cases) of mainly European (77%) and Asian (19%) ancestry	www.cardiogramplusc4d.org/, accessed on 26 May 2022
**Mediation analysis**			
Lipids	GLGC [18]	188,577 individuals of European ancestry	csg.sph.umich.edu/abecasis/public/lipids2013/, accessed on 26 May 2022
Body mass index	GIANT [15]	339,224 individuals of mainly European (95%) ancestry	portals.broadinstitute.org/collaboration/giant/index.php/GIANT_consortium, accessed on 26 May 2022
Waist-to-hip ratio	GIANT [15]	224,459 individuals of mainly European (94%) ancestry	portals.broadinstitute.org/collaboration/giant/index.php/GIANT_consortium
Smoking	TAGC [17]	74,053 individuals of European ancestry	www.med.unc.edu/pgc/results-and-downloads, accessed on 26 May 2022
Blood pressure	UK Biobank	317,756 individuals of European ancestry	http://www.nealelab.is/uk-biobank, accessed on 26 May 2022
Glycaemic traits	MAGIC [16]	46,186 non-diabetic individuals of European ancestry	www.magicinvestigators.org/, accessed on 26 May 2022
**Mendelian randomization analysis (BCAA to CAD clinical event)**			
Stroke	MEGASTROKE [13]	446,696 individuals of European ancestry	http://www.megastroke.org/ acknowledgments.html, accessed on 26 May 2022
Intracranial haemorrhage	FinnGen	202,568 individuals of European ancestry	https://finngen.gitbook.io/documentation/, accessed on 26 May 2022
Atrial fibrillation	GCST006414 [14]	65,446 individuals of mainly European (91%) ancestry	http://www.broadcvdi.org/, accessed on 26 May 2022
Cardiac arrest	FinnGen	73,969 individuals of European ancestry	https://finngen.gitbook.io/documentation/, accessed on 26 May 2022
heart attack/ myocardial infarction	UK Biobank	317,756 individuals of European ancestry	http://www.nealelab.is/uk-biobank, accessed on 26 May 2022
deep venous thrombosis	UK Biobank	317,756 individuals of European ancestry	http://www.nealelab.is/uk-biobank, accessed on 26 May 2022
Pulmonary embolism	UK Biobank	317,756 individuals of European ancestry	http://www.nealelab.is/uk-biobank, accessed on 26 May 2022

### 2.4. Development of a Genetic Instrument for Serum BCAA Concentrations

A total of 196 SNPs associated with serum BCAA (isoleucine, leucine, or valine) levels were identified in a meta-analysis of 24,925 individuals of European origin (Supplementary Data S1) [8]. These SNPs were associated with serum BCAA levels at a genome-wide significance level (*p* < 5.0 × 10^−8^) in the meta-analysis of the discovery and replication cohorts. If an SNP was associated with three BCAAs, we chose the strongest correlation as the association between the SNP and BCAA. We used linkage disequilibrium score regression to estimate the genetic correlation among the investigated traits [19]. The variants were defined as being independent of each other based on imperfect linkage disequilibrium (r^2^ < 0.8) in the 1000 Genome project data. In addition, two different protocols were established for IV screening approaches (r^2^ < 0.001 or lead SNP), respectively. MR analyses were performed based on these two sets of IV protocols.

### 2.5. Mediation Analysis

We analyzed available data for the association between the BCAA genetic instrument and risk factors to evaluate the potential mediating effects of risk factors in the association between BCAA levels and CAD risk. We used MR to obtain effect estimates of the exposure-outcome (i.e., BCAA levels and CAD risk), exposure-mediator (i.e., BCAA levels and risk factors), and mediator-outcome (i.e., risk factors and CAD risk) associations. The exposure–mediator and mediator–outcome associations could then be used to estimate the expected effect of BCAA levels on CAD risk, assuming that the association was mediated by the risk factors. This effect estimate was then contrasted with the observed exposure–outcome association to gain insights into the mediating effect of the putative mediator [20]. The calculation is described in detail in Text S1.

### 2.6. Meta-Analysis of Observational Studies of BCAA Levels and CAD Events

We conducted a systematic review and meta-analysis of published observational studies examining the association between BCAA levels and incident CVD. The details of the search strategies and the eligibility and exclusion criteria are presented in the Supplementary Material (Texts S2 and S3, and Figure S1). The results of seven studies were included in the meta-analysis using random effect models. Heterogeneity was quantified using the I^2^ statistic.

### 2.7. Gene Set Enrichment Analysis

We carried out a gene-based and gene set enrichment analysis of variant associations using MAGMA, implemented by FUMA. This analysis was performed using summary-level meta-analysis results. Gene- and pathway-based association tests were performed using study summary statistics in MAGMA v1.0680 in accordance with recommended procedures using reference files available at https://ctg.cncr.nl/software/magma (accessed on 24 February 2023) via the FUMA online server (http://fuma.ctglab.nl/, accessed on 24 February 2023). On top of the single gene level analyses, FUMA also provides information on association overrepresentation in sets of differentially expressed genes (DEG) to identify tissue specificity of prioritized genes. We conducted a gene-based association analysis to identify candidate genes associated with BCAA, followed by tissue enrichment analysis of BCAA-associated genes using gene expression data for 30 tissues from GTEx. The expression levels of genes and proteins in each tissue were obtained from https://tsomics.shinyapps.io/RNA_vs_protein/ (accessed on 24 February 2023) or http://genome-asia.ucsc.edu/ (accessed on 24 February 2023) [21].

### 2.8. Association of BCAA-Raising Loci with Other Phenotypes

We examined the association between BCAA-raising SNPs and MI induced by plaque rupture and thrombosis using a recently published GWAS meta-analysis (MI vs. restricted CAD-only) [22]. This meta-analysis included publicly available summary statistics for MI with 9,289,491 SNPs from the CARDIoGRAMplusC4D Consortium combined with GWAS results for MI in the UK Biobank. The odds ratio (OR) and associated 95% confidence intervals (CIs) were calculated for each potential BCAA-raising SNP.

To understand the BCAA-raising gene functions, we reported associations (*p* < 1 × 10^−5^) for sentinel variants with traits in the UK Biobank cohort using the MRBase PheWAS database (http://phewas.mrbase.org/, accessed 1 June 2022), which contains genome-wide association summary data for 4203 phenotypes measured in 361,194 unrelated individuals of European ancestry from the UK Biobank data. We queried GWAS data for eight traits related to pathological risk factors, endophenotypes, and related disease traits using summary-level data from the largest publicly available GWAS study. The possible metabolic regulatory factors leading to cardiovascular events due to the occurrence and progression of plaque have been focused on, including glucose and lipid metabolism (glycated hemoglobin, low-density lipoprotein-cholesterol (LDL-C), and body mass index (BMI) (from UK Biobank)), neuroendocrine regulation (renin [23], angiotensin-converting enzyme 2 [23], testosterone, and total choline (from UK Biobank)), and platelet count [24].

Specifically, it is known that the BCAA gene PPM1K affects diabetes [25], and defective BCAA catabolism disrupts glucose metabolism and sensitizes the heart to ischemia-reperfusion injury [26]. Neurohormonal activation is important in the development of cardiovascular diseases such as CAD, especially the renin-angiotensin system [27]. Several observational studies show an inverse association between serum testosterone concentration and adverse cardiovascular outcomes, metabolic syndrome, and mortality [28]. Ritchel et al. reported that central nervous system control of BCAAs may be mediated in part by vagal outflow [29]. In humans, elevated plasma levels of choline and products of its metabolism have been linked to the risk of CAD-related outcomes [30]. In addition, BCAA could regulate energy metabolism to promote the progression of cardiovascular disease, so BMI and lipids were included as candidate traits [26]. Finally, platelet dysfunction is an important cause of subsequent ischemic events in CAD, which may be regulated by BCAA [31]. We thus propose these eight traits as candidate pathologic features.

### 2.9. Hierarchical Agglomerative Clustering, Gene Interactions and Epigenetic Effects

Phenotypic clustering was estimated through agglomerative hierarchical clustering (AHC), calculating the Euclidean distance and using Ward’s agglomeration method [32]. We performed agglomerative hierarchical clustering to identify sets of genes sharing similar profiles. Hierarchical clustering based on effect size and direction of the association. We performed agglomerative hierarchical clustering across the top 17 independent loci using the directional Z -score (beta of continuous traits or log odds of disease risk divided by the standard error of the cross-trait associations) obtained from logistic regression analyses in each of the eight disease-specific GWASs. Where a sentinel variant was not available in any of the other trait summary results, a common proxy was used in place of the sentinel variant. We accounted for multiple testing at a family-wise error rate of 0.05 by Bonferroni correction for the eight traits tested per BCAA-raising locus (136 tests) and considered *p* < 3.7 × 10^−4^ (0.05/136) as the significance threshold for an association.

Gene interactions and networks were analyzed using the GeneMANIA prediction server (v3.5.1) (http://genemania.org, accessed on 24 February 2023) [33] and plotted using Cytoscape (v3.6.1). Pathway-based association testing was achieved by defining a biological pathway incorporating the gene targets of interest. In this study, we used the mQTL database (http://www.mqtldb.org, accessed on 24 February 2023) for this purpose, which reports SNP-methylation effect estimates obtained from the ARIES project (http://www.ariesepigenomics.org.uk/, accessed on 24 February 2023).

### 2.10. MR Analyses

The primary analysis was conducted using a random-effects inverse-variance-weighted method [34]. The secondary MR methods included MR-Egger [35], simple median, weighted median [36], MR–Robust Adjusted Profile Score (MR-RAPS) [37], and MR–Pleiotropy Residual Sum and Outlier (MR-PRESSO) [38] methods. The weighted median method can reduce the bias of valid inverse variances and is suitable for applications with multiple genetic analyses. The MR-Egger regression can detect and adjust for pleiotropy, but the precision of the estimates is low. The MR-RAPS approach was designed to identify and estimate confounded associations using weak genetic instrument variables. The MR-PRESSO method was used to identify potential outliers in multi-instrument summary-level MR testing and provide robust estimates with outlier correction.

### 2.11. Sensitive Analyses

To increase the reliability of MR results, we conducted multiple sensitivity analyses with respect to the MR tests to exclude possible biases (horizontal pleiotropy, i.e., variants included in the genetic instrument having an effect on the disease outside their effects on the exposure in MR) under different scenarios in the MR estimates [39]. Finally, a modified Cochran Q statistic and leave-one-out analysis were conducted to detect heterogeneity in the results [39]. As a result of these different methods used to compare the results, better agreement and higher reliability could be obtained.

### 2.12. Statistical Analyses

MR analyses were conducted using the TwoSampleMR R package [34]. All statistical tests were two-sided. All statistical analyses were conducted using Stata version 15.1 (Stata Corp, College Station, TX, USA) and R version 4.1 (R Foundation).

## 3. Results

### 3.1. MR Findings

Thirty-four SNPs were associated with BCAA concentrations after the screening. Seven genes related to confounder factors for CAD were not included because of the limitations of the MR method, and seventeen SNPs were finally included (Table 2; See Text S4 for details). The variation caused by these 17 SNPs accounted for 6.26% of the serum BCAA concentration. The proportion of variance in serum BCAAs explained by each SNP was calculated as r^2^ = 2 × minor allele frequency × (1 − minor allele frequency) × (β/SD)^2^, where the standard deviation (SD) = 1, because the data have been standardized. In the overall inverse-variance-weighted meta-analysis of the 17 SNPs, the OR of CAD per SD increase in genetically predicted serum BCAA was 1.08 (95%CI, 1.02–1.14; *p* = 0.007).

Concordant results were observed with the other MR methods. The significance was replicated with the weighted median (OR = 1.08; 95%CI, 1.01–1.16; *p* = 0.037), simple median (OR = 1.10; 95%CI, 1.02–1.19; *p* = 0.011), MR-RAPS (OR = 1.08; 95%CI, 1.02–1.14; *p* = 0.009), and MR-PRESSO (OR = 1.08; 95%CI, 1.04–1.12; *p* = 0.0003) approaches. The OR of the MR-Egger method was 0.99 (95%CI, 0.78–1.24) (intercept = 0.009 [−0.014, 0.033], *p* = 0.445) (Table 3 and Figure S2). We also conducted heterogeneity and pleiotropy tests to ensure the reliability of the MR results. Cochran Q tests for IVW (*p =* 0.99) indicated no heterogeneity in SNPs included in the study. No outliers were identified by the leave-one-out analysis (Figure S2). These results support the reliability of the MR results. The results of MR analysis showed the following relationships between the three BCAAs and CAD: leucine, OR = 1.08 (95%CI, 1.01–1.16, *p* = 0.036); isoleucine, OR = 1.13 (95%CI, 1.03–1.24, *p* = 0.006); and valine, OR = 1.07 (95%CI, 1.00–1.14, *p* = 0.058) (Table S1 and Figure S3). The results showed that the partial association of BCAA with MR of cardiovascular disease showed a correlation when strict LD cut-offs were used (r^2^ < 0.001 or lead SNP), but the direction remained consistent (Tables S2 and S3).

### 3.2. Mediating Effects

In conventional MR analyses, a genetic predisposition towards higher serum BCAA levels was associated with lower high-density lipoprotein (HDL) cholesterol (*p* = 0.001), higher systolic blood pressure (SBP) (*p* < 0.001), higher diastolic blood pressure (DBP) (*p* < 0.001), and type 2 diabetes mellitus (*p* < 0.001), but not with LDL-C, total cholesterol, triglycerides, fasting glucose, fasting insulin, insulin resistance, body mass index, waist-to-hip ratio, or smoking (*p* > 0.05) (Table S4). Given that previous MR studies found no causal relationship between HDL and CAD [40], we only analyzed the mediating effects of SBP, DBP, and type 2 diabetes, which were identified as important mediators involved in the regulation of CAD by BCAAs (34.2%, 15.2%, and 33.5%, respectively) (Table 4). Surprisingly, blood pressure appeared to exhibit a stronger mediating effect than diabetes in terms of both proportion and effect value (SBP: beta [se] = 0.566 [0.091]; DBP: beta [se] = 0.581 [0.076]; type 2 diabetes mellitus: beta [se] = 0.110 [0.028]).

### 3.3. Meta-Analysis Findings

A meta-analysis of observational studies revealed that high levels of BCAAs were associated with a high risk of developing CAD (risk ratio [RR] = 1.18 [95%CI, 1.11–1.24 from 7 studies with 38,975 individuals; 4685 CAD cases/events; I^2^ 44.3%; random-effects meta-analysis]), after adjustment for age, sex, smoking status, BMI, blood pressure, and lipids. The RR was 1.31 (95%CI, 1.10–1.51) in three case–control studies and 1.14 (95%CI, 1.10–1.17) in three prospective cohort studies. The specific results are shown in the Supplementary Materials (Text S3, Table S5 and Figure S4).

### 3.4. BCAA Linked to Ischemic CVD Events

**Figure 2:**
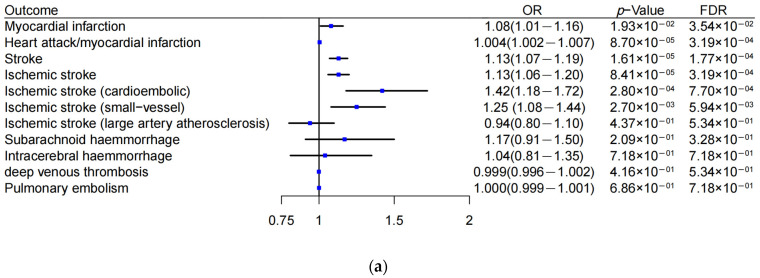

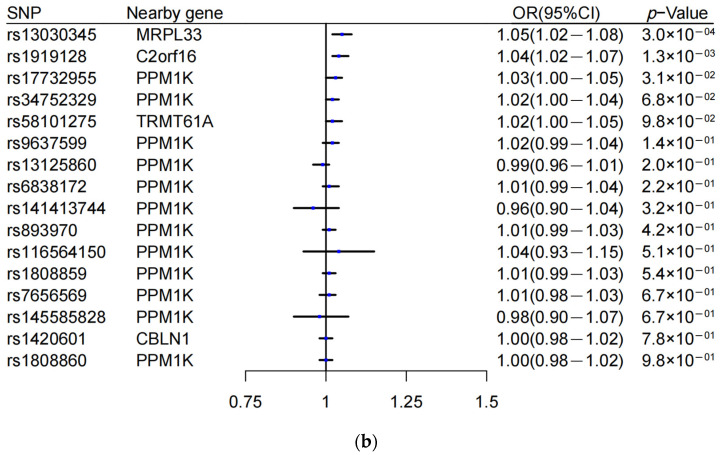
Branched-chain amino acid (BCAA)-raising genes and risk of ischemic events. (**a**) Mendelian randomization estimates of BCAAs in seven cardiovascular disease-related clinical events. (**b**) Preferential association of BCAA-raising genes with myocardial infarction in the presence of atherosclerosis. Blue dots represent the OR value. Lines represent 95% credible intervals. OR, odds ratio. FDR, false discovery rate. SNP, single-nucleotide polymorphism.

We applied MR analyses to investigate the effects of BCAAs on seven CVD clinical events including ischemic events (MI, heart attack, stroke, and its four subtypes (including ischemic stroke, cardioembolic ischemic stroke, small-vessel and large artery atherosclerosis ischemic stroke), deep venous thrombosis, and pulmonary embolism) and bleeding events (subarachnoid hemorrhage, intracerebral hemorrhage) (Figure 2a). Using conventional MR, 5 of the 11 analyses provided evidence of an impact of BCAAs on the risk of ischemic events based on a false discovery rate (FDR) <5%. Stronger evidence was found for the risk of ischemic events due to plaque rupture and thrombus: stroke risk (OR = 1.13, 95%CI, 1.07–1.19, *p* = 1.61 × 10^−5^), MI (OR = 1.08, 95%CI, 1.01–1.16, *p* = 1.93 × 10^−2^), and heart attack (OR = 1.004, 95%CI, 1.002–1.007, *p* = 8.70 × 10^−5^). The effect remained robust after correcting the multivariable analyses for FDR (FDR < 0.05). In addition, it did not show that BCAAs were causally linked to the risk of simple thrombosis (including deep venous thrombosis and pulmonary embolism) or bleeding events (*p* > 0.05). All MR estimates derived in these analyses are shown in Figure 2a.

### 3.5. Preferential Associations of MRPL33 and C2orf16 Loci with MI in the Presence of Atherosclerosis

Evidence from the large-scale human genetic analysis was consistent with a causal role of BCAA metabolism in ischemic CVD events due to plaque rupture and thrombus formation. Therefore, we assumed that BCAAs might be involved not only in plaque initiation and progression (chronic impairment factor in CAD) but also in plaque rupture and thrombosis (trigger for ischemic events). We evaluated the relationship between BCAA-raising genes (*PPM1K*, *TRMT61A*, *CBLN1*, *MRPL33*, and *C2orf16*) and the risk of MI (GWAS data with MI vs. CAD only). Two loci were related to plaque rupture: rs13030345 (OR = 1.05, 95%CI, 1.02–1.08; *p* = 3 × 10^−4^) in *MRPL33* and rs1919128 (OR = 1.04, 95%CI, 1.02–1.07, *p* = 1.3× 10^−3^) in *C2orf16*, while the other loci were not related to the risk of MI (*p* > 0.05) (Figure 2b).

### 3.6. Functional Analysis of BCAA-Raising Genes

Tissue-specificity analysis across tissue types from the GTEx project identified the organs with the greatest gene expression enrichment (Figure S5). Only *PPM1K* was specifically expressed in the heart, while the other three genes were mainly expressed in the central nervous system and the endocrine system, including the hypothalamus (*CBLN1*), adrenal gland (*MRPL33*), and testis (*C2orf16*). In addition, *TRMT61A* was mainly expressed in the esophagus (Figure S5)**.** In addition to the single gene level analyses, we also identified tissue specificity of prioritized genes by looking at overrepresentation in sets of differentially expressed genes (DEG) (Figure S6**)**. DEG for each tissue was calculated in FUMA. We found the colon, salivary gland, blood, bladder, and blood vessels to be the top five tissues with the most DEG.

**Figure 3:**
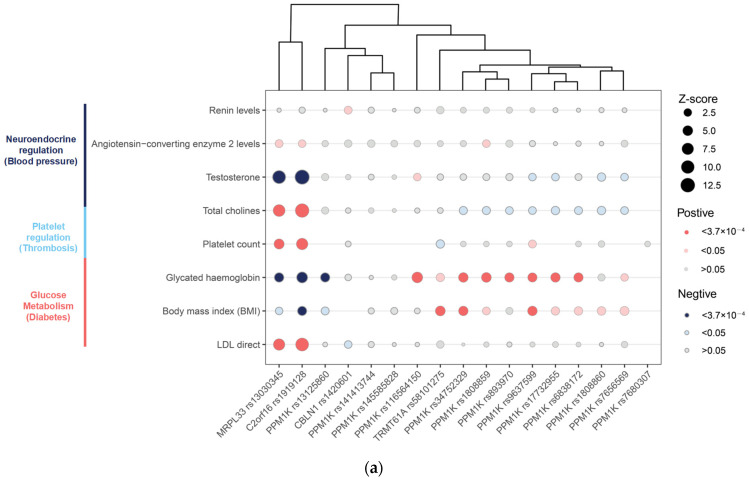

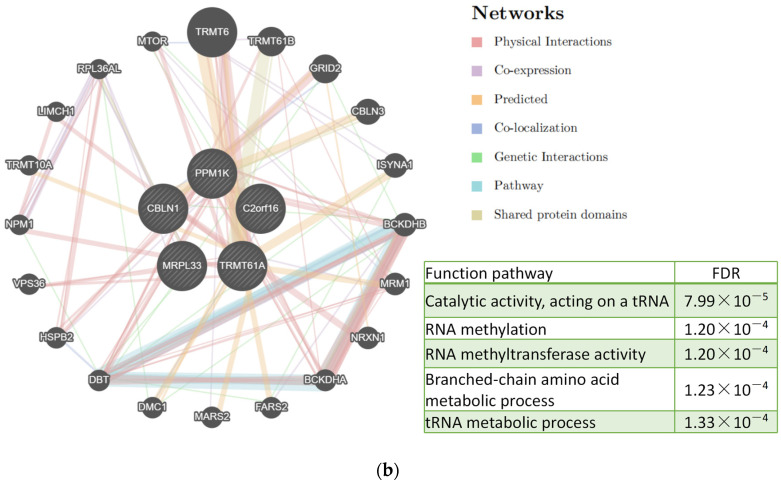
Potential functional impacts of branched-chain amino acid (BCAA) loci. (**a**) Associations of BCAA risk variants with traits relating to pathological indicators. Bubble plot shows associations between identified BCAA loci and pathological indicators using summary estimates from UK Biobank and published genome-wide association summary statistics. Bubble size represents the absolute Z-score for each trait, with direction oriented towards the BCAA risk allele. Red/blue indicates positive/negative cross-trait association (i.e., increase/decrease in risk or increase/decrease in continuous trait). We accounted for a family-wise error rate at 0.05 by Bonferroni correction for the eight traits tested per BCAA locus (*p* < 3.7 × 10^−4^); traits meeting this threshold of significance for the association are indicated by darker shading. (**b**) Gene network of the susceptibility pathways and GO analysis of related genes (top5 pathway). Each node is a gene, which was the selected gene in our GWAS pathway analysis. The connecting lines are drawn if the two genes have a relationship, such as co-expression, shared protein domain, physical interactions, co-localization, or pathway. The thickness of the lines represents the degree of similarity between two genes. Gene network is produced using GeneMANIA. LDL, low-density lipoprotein.

We then investigated the associations between the BCAA-raising genes and plaque rupture/thrombosis traits to provide potential insights into the etiology. We first queried the large database of genetic associations in the UK Biobank (http://www.nealelab.is/uk-biobank, accessed on 26 May 2022) and identified several biomarkers and disease associations at each locus. *PPM1K* has been found to be an important regulator of glucose metabolism and diabetes, which also revealed the casual association for type 2 diabetes [25]. The functions of *MRPL33* and *C2orf16* as solute carriers are not entirely known, but they have been reported to involve neuroendocrine diseases, such as autism spectrum disorder and type 2 diabetes [41,42]. We tested for associations of the BCAA-raising genes with eight putative risk factors of plaque rupture and thrombus, including neuroendocrine, platelet, and glucose metabolism factors, using GWAS summary data (Figure 3a). Sites on *PPM1K* and *TRMT61A* were mainly related to hemoglobin levels. *CBLN1* was weakly correlated with renin levels, while *MRPL33* and *C2orf16* were correlated with angiotensin, testosterone, total choline, platelet count, glucose metabolism, BMI, and LDL, which differed significantly from the *PPM1K* gene-phenotype. Elevated plasma levels of choline and products of its metabolism have been linked to the risk of MI-related outcomes in humans [43].

Gene interaction analysis demonstrates high network connectivity between most of the identified BCAA genes (Figure 3b). By way of physical interactions, co-expression, prediction, colocalization, genetic interactions, pathways, and common protein domains, nodes in the network demonstrated that certain genes were linked to TRMT61A and PPM1K. GO results analysis showed that it mainly enriched to tRNA catalytic activity, RNA methylation, and branched-chain amino acid metabolic process. Therefore, we focused on exploring the relationship between these BCAA SNPs and DNA methylation. Seven out of the seventeen loci had at least one genetic variant (mQTL), in cis and/or trans, associated with methylation levels (Additional file: Supplementary Data S2).

**Figure 4:**
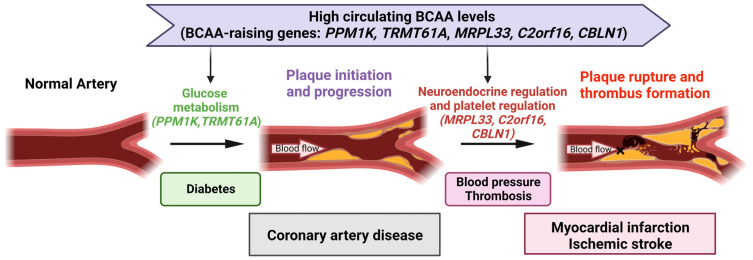
Schematic summary of a causal link and potential mechanisms of branched-chain amino acids (BCAAs) in cardiovascular disease (CVD). High circulating BCAA levels may influence CAD and subsequent plaque rupture events via two distinct pathways: glucose metabolism disorder (as shown by *PPM1K* and *TRMT61A*) through processes leading to chronic vulnerability to CAD and neuroendocrine dysregulation (as shown by *CBLN1*, *MRPL33*, and *C2orf16*) affecting hemodynamic factors and platelets through neuroendocrine factors, which in turn affect subsequent plaque rupture and thrombosis in CAD, triggering ischemic events. CAD, coronary artery disease. BCAA, branched-chain amino acids. Figure created with BioRender (biorender.com, accessed on 24 February 2023).

Collectively, these results suggested that high circulating BCAA levels can influence CAD and subsequent plaque rupture events via two distinct pathways: glucose metabolism disorder (as shown by *PPM1K* and *TRMT61A)* through processes leading to chronic vulnerability to CAD and neuroendocrine dysregulation (as shown by *CBLN1*, *MRL33*, and *C2orf16)* affecting hemodynamic factors and platelets through neuroendocrine factors, which in turn affect subsequent plaque rupture and thrombosis in CAD, triggering ischemic events. Taken together, these data provide supportive, functional evidence that genetically elevated BCAA levels may contribute to an increased risk of plaque rupture and thrombosis not only through glucose metabolism but, more likely, through neuroendocrine mechanisms (Figure 4).

## 4. Discussion

The results of this MR study supported a potential causal link between elevated BCAA levels and an increased risk of CVD. The principal findings confirmed the results of several observational studies, suggesting that elevated serum BCAA levels may translate into an increased risk of CAD. The findings were robust in sensitivity analyses with different instruments and statistical models. Complementary analyses provided further evidence of the adverse effects of genetically predicted serum BCAA levels on the risks of ischemic CVD events caused by plaque rupture and thrombosis. In addition to chronic vulnerability to CAD, the results also suggested that BCAA may affect the neuroendocrine promotion of plaque rupture and thrombosis through platelet and hemodynamic factors (such as blood pressure), leading to ischemic CVD events.

The circulating pool of free BCAAs is determined by a balance between their input (e.g., diet and proteolysis) and output (e.g., protein synthesis and catabolism for energy) [44]. BCAAs are essential amino acids that can only be obtained from external food sources, and their homeostasis must therefore be maintained by catabolism. The first two steps in the pathway are common to all three BCAAs: the first reaction, catalyzed by branched-chain aminotransferase (BCAT), is a reversible transamination to form branched-chain α-keto acids (BCKAs), and the second reaction, catalyzed by the branched-chain α-keto acid dehydrogenase (BCKD) complex, is an irreversible oxidative decarboxylation of BCKAs and is the rate-limiting step in the overall BCAA catabolic pathway (Figure S7) [45]. GWAS analysis indicated that most BCAA-related SNPs were located in the *PPM1K* gene, in which loss-of-function mutations result in impaired BCKD activity [46]. Impaired BCKD signaling, in turn, contributes to disturbed catabolism of BCAAs and their accumulation in circulation. The genetic variation found in this study may thus reflect impaired catabolism of BCAAs, leading to higher circulating levels. GWAS data also showed that BCAT-related genes were not significantly associated with circulating BCAAs. This might be because of the reversible steps of BCAT mediation or because its genes are more uniformly distributed across the population. The instrumental variables used in the current study provide the key to adjustments in BCAA concentrations.

BCAA supplementation may improve energy expenditure [47]; however, increased circulating levels of BCAAs are also associated with diabetes, coronary heart disease, and heart failure [48]. Yoneshiro et al. proposed a unique, non-mutually exclusive theory to explain this apparent contradiction. Following cold exposure in mice and humans, brown adipose tissue (BAT) actively utilized BCAAs in the mitochondria for thermogenesis and promoted systemic BCAA clearance. In turn, a BAT-specific defect in BCAA catabolism attenuated systemic BCAA clearance, BAT fuel oxidation, and thermogenesis, leading to glucose intolerance [49]. Similarly, the increase in circulating BCAAs caused by genetic mutations may be due to catabolism disorders. Supplemented BCAAs may thus be unable to participate effectively in improving the body’s energy metabolism.

The current MR analysis of BCAA and CVD risk factors indicated that BCAAs were related to SBP, DBP, and type 2 diabetes. The relationship between circulating BCAA levels and coronary heart disease was unlikely to be explained by the main lipids because genetically higher BCAA levels are unrelated to cholesterol and triglyceride levels, and existing MR studies have found an unclear relationship between increased HDL and CVD [50,51]. Mediation analysis suggested that the effect of BCAAs on CVD was partly mediated by blood pressure and type 2 diabetes, and GWAS analysis of BCAA concentrations confirmed that BCAA-related genes affecting CVD were related to disorders of glucose metabolism. The strongest signal was located in *PPM1K*, which encodes BCKD and mitochondrial phosphatase. Moreover, mutation or loss of *PPM1K* leads to maple diabetes. Luca et al. found a causal relationship between BCAAs and type 2 diabetes, with the strongest signal also located on the *PPM1K* gene [25]. Some studies suggested that insulin resistance in endothelial cells was the main cause of coronary atherosclerosis [52], and BCAAs have been shown to upregulate glucose transporters and activate insulin secretion [53,54]. BCAA catabolism disorders lead to impaired mitochondrial activity and redox capacity, increased glucose, glycolysis intermediates, and glucose-derived sugars [44]. Long-term accumulation of BCAAs will lead to down-regulation of the biological exposure pathway of glucosamine, including the inactivation of pyruvate dehydrogenase, making the cardiovascular system vulnerable to damage [26].

Interestingly, the current results showed that BCAAs might also affect ischemic events caused by plaque rupture or thrombosis through the neuroendocrine system, especially via the regulation of blood pressure and platelets. Tissue expression and putative gene function analysis showed that three BCAA-related genes were involved in the modulation of neuroendocrine circuits. The *CBLN1* gene encodes a cerebellar precursor-specific protein that acts primarily on post-synaptic Purkinje fibers, which is a component of the signaling pathway [55]. *CBLN1* is a novel appetite-stimulating peptide related to renin expression that may be mediated by hypothalamic neuropeptide [56], which is a sympathetic neurotransmitter related to hypertension and a variety of CVDs [57]. *MRPL33* has been causally associated with a higher risk of autism spectrum disorder [41] and with angiotensin-converting enzyme 2 and testosterone levels. *C2orf16* is mainly expressed in the testis and has also been associated with angiotensin-converting enzyme 2 and testosterone. In addition, *MRPL33* and *C2orf16* were also correlated with choline and platelet count, while choline, which is involved in central and peripheral sympathetic innervation, participates in neuroendocrine regulation and is closely related to platelet activation [58,59]. Recent studies have examined the relationship between BCAAs and the central nervous system. Ritchel et al. reported that central nervous system control of BCAAs may be mediated in part by vagal outflow [29]. Recent articles showed that plaques could be regulated by the brain via the central nervous system, whereas BCAAs may also participate in CVD as a neuroregulatory signal [29,60]. In general, these three genes may affect the sympathetic renin–angiotensin axis, which may provide an explanation for the mediating roles of hypertension and platelets. Functional analysis of these genes suggested that BCAAs may promote CVD via neuroendocrine pathways, and further studies are needed to clarify the molecular mechanisms linking BCAA metabolism to an increased risk of CVD.

Many conventional observational studies have confirmed a longitudinal association between high BCAA concentrations and increased risks of many CVDs [61,62]. The current MR analysis suggested that the effect of BCAAs on CVD could be generalized to cardiovascular ischemic outcomes caused by plaque rupture or thrombosis. This finding included evidence for a total effect of BCAA on the risk of ischemia CVD events later in life, corroborating recent basic studies suggesting that the increased event risk may be explained by defective BCAA catabolism determined by genetic switching [26]. Most studies have consistently reported that BCAA mediators influence alterations in glucose, oxidative stress, and inflammation, leading to endothelial dysfunction and atherosclerosis [7,63]. Atherosclerosis is the main cause of CVD and interacts synergistically with hypertension and diabetes, with these three factors aggravating each other [64]. Regarding ischemic events, gene function analysis suggested that BCAAs might affect hemodynamics and platelets through neuroendocrine regulation and might trigger plaque rupture or thrombosis. Furthermore, recent studies found that BCAA catabolism promoted the risk of thrombosis by enhancing tropomodulin-3 propionylation in platelets, which may act as a trigger for ischemic events [31]. This was also confirmed by the current finding that *MRPL33* and *C2orf16* affected platelet function. Overall, the causal effect may involve two pathophysiological pathways, including glucose metabolism (*PPM1K* and *TRMT61A*) related to plaque progression and the newly discovered neuroendocrine disorders (*CBLN1*, *MRPL33*, and *C2orf16*) related to plaque rupture and thrombosis (Figure 4).

BCAAs may increase the risk of CVD via several other mechanisms. Leucine may inhibit endothelial cell synthesis, thus promoting endothelial cell dysfunction and increasing the risk of atherosclerosis [65]. *TRMT61A* encodes a methyltransferase that plays an important role in tRNA synthesis [66]. High concentrations of BCAAs have been shown to upregulate mammalian targets of rapamycin activity, affecting cardiac protein, lipid, glucose, and nucleotide metabolism and autophagy [67]. BCAAs/BCKA is known to inhibit the transport and utilization of pyruvate and fatty acids, thus increasing the sensitivity of cardiovascular cells to chemical damage. Pyruvate and fatty acids are the main sources of energy in the heart and are in persistently high demand by heart cells, and elevated BCAAs/BCKA may thus affect normal cardiovascular energy homeostasis leading to increased cell load, vasoconstriction, and accelerated heart failure [68]. Higher BCAA/BCKA levels may also have a potentially negative cardiovascular effect by regulating the mitochondrial electron transport chain and mitochondrial permeability transition pores [69].

BCAA metabolism is closely linked to other metabolic pathways, and changes in this metabolic pathway may, therefore, only influence CVD risk in certain metabolic contexts. Lipid metabolism-related genes also affect blood levels of metabolites other than BCAAs, including total cholesterol, LDL, and triglycerides, which have been associated with a higher incidence of CVD in observational studies [70,71]. The effects of BCAA-related lipid metabolism genes on lipids and coronary heart disease might mask the relationship between BCAAs and coronary heart disease. To avoid violating the MR hypothesis, we excluded horizontal genes from the current study. However, the results indicated that activation of BCAAs by specific pathways may increase the risk of CVD and subsequent ischemic events.

This study had several limitations. Samples contributing to the study-level MR were also included in the much larger CAD GWAS used for the summary-level MR, implying some dependence between the results, even though the methodologies were different. The predominantly European ancestry of the subjects also limited the generalizability of the results to other ethnic groups. In addition to the vertical pleiotropy considered above, there was evidence for horizontal pleiotropy that was addressed through current best practices for MR sensitivity analysis, all of which supported the primary conclusions. However, as with all MR studies, we could not address unobserved pleiotropy. In addition, a potential non-linear association between serum BCAA levels and CAD could not be evaluated. Limited by the summary data of BCAA GWAS, we found that the recent MR analysis of BCAA also did not carry out non-linear analysis, including the MR analysis of BCAA and type 2 diabetes, insulin resistance and stroke [25,72,73]. Furthermore, a further limitation of MR methods is that the effects of the exposure or mediator are non-linear. Although some methods are emerging for carrying out MR analysis with non-linear exposures [74,75], these methods have not yet been extended to MR mediation analyses. Current MR methods for mediation analysis will assume a linear association between exposure and outcome. In addition, a conservative approach was adopted, and at the cost of reducing power, the use of loci specific to each phenotypic trait (r^2^ < 0.001 or lead SNP) showed a weak correlation. Furthermore, we only considered changes in BCAA concentrations as a result of genetic factors, which only accounted for a small proportion of the changes in BCAA levels. Although we evaluated blood pressure and type 2 diabetes as mediators of the causal link between BCAAs and CAD, BCAA metabolism is a complex process that is closely related to other pathways, and other unknown pathways and environmental factors will thus also affect CAD. BCAA-SNPs are involved in methylation maintenance. The role of epigenetic effects in modifying the effects of the BCAA on CAD deserves future research. Further investigations are warranted to illuminate the underlying pathological mechanisms by which BCAAs affect coronary heart disease, including their possible involvement in neuroendocrine regulation.

## 5. Conclusions

Evidence from this large-scale human genetic and metabolomic study was consistent with a causal role of BCAA metabolism in CVD and subsequent ischemic events. The causal effect was partly mediated by blood pressure and type 2 diabetes. Functional analysis of the BCAA-raising gene signatures revealed that the causal effect might involve two pathophysiological pathways, including glucose metabolism (*PPM1K* and *TRMT61A)* related to plaque progression, and the newly discovered neuroendocrine disorders (*CBLN1*, *MRPL33*, and *C2orf16*) related to plaque rupture and thrombosis. We speculate that future analyses of BCAA in relation to neuroendocrine system function may yield additional insights into the genetic architecture of CVD to inform new approaches to its prevention and treatment.

## Figures and Tables

**Table 2:** Characteristics of branched-chain amino acid-associated genetic variants.

SNP	Nearby Gene	Chromosome	Effect/Other Alleles	Effect Allele Frequency	Beta(Se) of BCAAs Level Per Alleles^1^	*p*-Value ^1^	OR(95%CI) for CAD Per Alleles ^2^	*p*-Value ^2^
rs116564150	PPM1K	4	A/G	0.013	0.31(0.05)	5.79 × 10^−12^	1.07(0.97,1.18)	0.188
rs145585828	PPM1K	4	A/C	0.016	−0.25(0.04)	2.06 × 10^−9^	1.01(0.93,1.11)	0.753
rs141413744	PPM1K	4	C/T	0.037	−0.20(0.03)	1.24 × 10^−14^	1.02(0.95,1.11)	0.555
rs1808860	PPM1K	4	C/T	0.436	0.11(0.01)	1.95 × 10^−31^	1.01(0.99,1.03)	0.458
rs34752329	PPM1K	4	A/G	0.369	0.11(0.01)	5.69 × 10^−30^	1.01(0.99,1.03)	0.286
rs6838172	PPM1K	4	A/G	0.320	0.11(0.01)	5.68 × 10^−27^	1.01(0.99,1.03)	0.348
rs7680307	PPM1K	4	C/T	0.259	0.11(0.01)	5.87 × 10^−20^	1.01(0.99,1.04)	0.421
rs9637599	PPM1K	4	C/A	0.470	0.11(0.01)	7.64 × 10^−36^	1.00(0.98,1.02)	0.757
rs13125860	PPM1K	4	A/T	0.389	−0.10(0.01)	1.69 × 10^−25^	1.00(0.98,1.02)	0.904
rs17732955	PPM1K	4	T/C	0.295	0.10(0.01)	1.09 × 10^−22^	1.02(1.00,1.04)	0.116
rs1808859	PPM1K	4	C/T	0.391	0.10(0.01)	5.92 × 10^−26^	1.00(0.98,1.02)	0.696
rs58101275	TRMT61A	14	G/A	0.790	0.09(0.02)	9.87 × 10^−10^	1.01(0.99,1.04)	0.222
rs893970	PPM1K	4	C/T	0.447	0.09(0.01)	2.98 × 10^−19^	1.00(0.98,1.02)	0.689
rs13030345	MRPL33	2	T/G	0.191	0.07(0.01)	4.52 × 10^−9^	1.01(0.98,1.03)	0.488
rs1420601	CBLN1	16	C/T	0.400	0.07(0.01)	3.63 × 10^−8^	1.01(0.99,1.03)	0.244
rs1919128	C2orf16	2	G/A	0.275	0.07(0.01)	1.18 × 10^−10^	1.01(0.99,1.03)	0.422
rs7656569	PPM1K	4	A/C	0.202	0.07(0.01)	6.74 × 10^−9^	1.01(0.98,1.04)	0.464

^1^ Summary statistics per effect allele for serum branched-chain amino acid (BCAA) levels obtained from a genome-wide association meta-analysis. ^2^ Effect size estimate (β coefficient, measured as log odds ratio of coronary artery disease [CAD] per additional BCAA-raising allele, and standard error) and *p*-value for each single-nucleotide polymorphism in CAD were obtained from the CARDIoGRAMplusC4D consortium.

**Table 3:** Mendelian randomization effect size for branched-chain amino acid-associated single-nucleotide polymorphisms in coronary artery disease.

Method ^1^	Beta	Se	OR(95%CI)	*p*-Value
Inverse variance weighted	0.076	0.028	1.08(1.02,1.14)	0.007
Weighted median	0.078	0.038	1.08(1.01,1.16)	0.037
Simple median	0.097	0.040	1.10(1.02,1.19)	0.011
MR-RAPS	0.077	0.029	1.08(1.02,1.14)	0.009
MR-PRESSO	0.076	0.017	1.08(1.04,1.12)	0.0003

^1^ Mendelian randomization (MR)-Egger regression OR = 0.99 (0.78, 1.24), *p* = 0.913; intercepts: 0.009 (−0.014 to 0.033), *p* = 0.445.

**Table 4:** Estimates of mediating effects of type 2 diabetes mellitus, systolic blood pressure, and diastolic blood pressure on the association between branched-chain amino acids and coronary artery disease using the inverse-variance-weighted method.

	Beta(Se) ^1^	*p* Value ^1^	Mediation Effect (%) ^2^
Systolic blood pressure	0.566(0.091)	<0.001	34.2%
Diastolic blood pressure	0.581(0.076)	<0.001	15.2%
Type 2 diabetes	0.110(0.028)	<0.001	33.5%

^1^ Mendelian randomization estimates of effects of systolic blood pressure, diastolic blood pressure, and type 2 diabetes on coronary artery disease. ^2^ Mediating effect as a proportion of total effect.

## Data Availability

The summary data in this study are publicly available and presented in Table 1 and in the Supplementary Material.

## Supplementary Materials

The following supporting information can be downloaded at: https://www.mdpi.com/article/10.3390/metabo13030403/s1, Text S1: Mediation analysis; Text S2: Methods for systematic review and meta-analysis; Text S3: Systematic review and meta-analysis of the association between BCAA levels and incident CAD. Text S4: BCAA-associated SNPs with risk factors are not a suitable tool for MR analysis; Table S1. Branched-chain amino acid-associated genetic variants screening data after applying a strict linkage disequilibrium cut-off (r^2^ < 0.001 or lead SNP).; Table S2: Mendelian randomization estimates of the association between genetically predicted increases in serum leucine, isoleucine, and valine levels and coronary artery disease; Table S3. Mendelian randomization effect size for branched-chain amino acid-associated single-nucleotide polymorphisms in cardiovascular disease. Table S4: Associations between genetically predicted serum branched-chain amino acid levels and cardiometabolic risk factors; Table S5: Study characteristics; Figure S1: Workflow for systematic review of published observational studies examining the association between branched-chain amino acid levels and incident coronary artery disease; Figure S2: Scatter and leave-one-out plots demonstrating influential outliers in Mendelian randomization of branched-chain amino acids and coronary artery disease risk; Figure S3: Mendelian randomization (MR) estimates of the association between genetically predicted increases in serum leucine, isoleucine, valine levels and coronary artery disease (CAD); Figure S4: Meta-analysis of observational associations between baseline branched-chain amino acid levels and incident coronary artery disease; tissue enrichment analysis based on gene expression; Figure S5. Gene expression enrichment of BCAA loci in GTEx tissues. Figure S6. Tissue enrichment analysis based on gene expression. Figure S7. Summary of the catabolism pathways of BCAAs. Supplementary Data S1: Characteristics of all branched-chain amino acid-associated genetic variants. Supplementary Data S2: mQTL analysis and candidate SNPs identification for BCAA.

## Abbreviations

BCAA	branched amino acid
MR	Mendelian randomization
CVD	cardiovascular disease
CAD	coronary artery disease
MI	myocardial infarction
SNP	single-nucleotide polymorphisms
GWAS	genome-wide association studies
GTEx	Gene-Tissue Expression Project
BCKD	branched-chain α-keto acid dehydrogenase
ROS	reactive oxygen species
OR	odds ratio
CI	confidence interval
