# Supplementary material for: Mendelian Randomization Analysis Provides Insights into the Pathogenesis of Serum Levels of Branched-Chain Amino Acids in Cardiovascular Disease
Source: Metabolites. 2023 Mar 9;13(3):403. doi: 10.3390/metabo13030403 (PMC10059809; doi:10.3390/metabo13030403)
Supplement: Supplementary file 1 [file metabolites-13-00403-s001.zip › Supplemental_20230308_clean.pdf]

Supplemental Material

Mendelian randomization analysis provides insights into the pathogenesis of serum levels of branched-chain amino acids in cardiovascular disease

Text S1. Mediation analysis 3

Text S2. Methods for systematic review and meta-analysis 4

Text S3. Systematic review and meta-analysis of the association between BCAA levels and incident CAD 6

Text S4. BCAA-associated SNPs with risk factors are not a suitable tool for MR analysis.... 7

Table S1. Mendelian randomization estimates of the association between genetically predicted increases in serum leucine, isoleucine, and valine levels and coronary artery disease 8

Table S2. Branched-chain amino acid-associated genetic variants screening data after applying a strict linkage disequilibrium cut-off (r^2<0.001 or lead SNP) 9

Table S3. Mendelian randomization effect size for branched-chain amino acid-associated single-nucleotide polymorphisms in cardiovascular disease after applying a strict linkage disequilibrium cut-off (r^2<0.001 or lead SNP). 10

Table S4. Associations between genetically predicted serum branched-chain amino acid levels and cardiometabolic risk factors. 11

Table S5. Study characteristics 12

Figure S1. Workflow for systematic review of published observational studies examining the association between branched-chain amino acid levels and incident coronary artery disease 13

Figure S2. Scatter and leave-one-out plots demonstrating influential outliers in Mendelian randomization of branched-chain amino acids and coronary artery disease risk 14

Figure S3. Mendelian randomization (MR) estimates of the association between genetically predicted increases in serum leucine, isoleucine, valine levels and coronary artery disease (CAD) 15

Figure S4. Meta-analysis of observational associations between baseline branched-chain amino acid levels and incident coronary artery disease 17

Figure S5. Gene expression enrichment of BCAA loci in GTEx tissues 17

Figure S6. Tissue enrichment analysis based on gene expression 21

Figure S7. Summary of the catabolism pathways of BCAAs 22

Text S1. Mediation analysis

We used the inverse-variance-weighted MR method to calculate the associations between systolic blood pressure (SBP), diastolic blood pressure (DBP), and CAD. The instrumental variables of SBP and DBP came from UK Biobank, and 157 SNPs for SBP and 172 SNPs for DBP were included ($P < 5\times10^{-8}$ and $r^2 < 0.001$). We used available data on the association between the BCAAs genetic instrument and type 2 diabetes[1] in a mediation analysis to evaluate the potential mediating effect of type 2 diabetes in the association between BCAA levels and CAD risk. We used MR to obtain effect estimates of the exposure-outcome (i.e., BCAA levels and CAD risk), exposure-mediator (i.e., BCAA levels SBP, DBP, and type 2 diabetes), and mediator-outcome (i.e., SBP, DBP, type 2 diabetes, and CAD risk) associations. The exposure-mediator and mediator-outcome associations could then be used to estimate the expected effect of BCAA levels on CAD risk assuming that this association was mediated by SBP, DBP, and type 2 diabetes. This effect estimate could then be contrasted with the observed exposure-outcome association to gain insights into the mediating effect of the putative mediator. We finally divided the mediated effect by the total effect to estimate the proportion mediated, as reported previously.[2,3]

Text S2. Methods for systematic review and meta-analysis

1) Systematic review of the literature

We conducted a systematic review of published observational studies examining the relationship between BCAA levels and incident CAD. We searched MEDLINE and Embase up to 2 December 2018 using a pre-specified, computer-based, inclusive search strategy (detailed below). The titles, abstracts, and full articles were screened to identify observational studies that investigated baseline plasma BCAA levels in relation to the risk of incident CAD (Figure S1).

2) Inclusion and exclusion criteria

The study inclusion criteria were: (1) study population adults; (2) papers examined the relationship between BCAA and CAD; (3) study endpoints were the incidence of CAD, with ORs or HRs, or ORs or HRs were calculable from the data provided; (5) concentration of BCAAs alone or change in concentrations; and (6) studies in Chinese or English literature. The exclusion criteria were: (1) studies of mechanisms; (2) animal studies; (3) repeat publications; and (4) literature that are not available.

3) Data extraction and literature bias risk assessment

Two investigators jointly selected the studies that potentially met the inclusion criteria and read the full texts to assess their eligibility. A literature-extraction database was then established. Depending on the type of study, we used the Newcastle-Ottawa Scale (NOS) scale to assess bias in the included cohort studies and case-control studies, including the choice of subjects (4 points), comparability (2 points), and exposure (3 points). Scores ≥7 were classified as low bias risk, 6 as moderate bias risk, and ≤5 as high bias risk. Cross-sectional study quality was assessed according to the Agency for Healthcare Research and Quality (AHRQ).

4) Search terms and strategies

Publication database: MEDLINE

#1 BCAA OR branched chain amino acid OR valine OR isoleucine OR leucine

#2 cardiovascular OR heart OR MI OR myocardial infraction OR CAD OR HF OR ischemic OR IHD OR CVD OR ASCVD OR AMI

#3 "Humans"[Mesh]

Search: #1 AND #2 AND #3

Restrictions: None

Result: 5,300

Publication database: Embase

#1 (BCAA OR branched chain amino acid OR valine OR isoleucine OR leucine).af

#2 (cardiovascular OR heart OR MI OR myocardial infraction OR CAD OR HF OR ischemic OR IHD OR CVD OR ASCVD OR AMI).af

Search: #1 AND #2

Restrictions: Human

Result: 4,474

Text S3. Systematic review and meta-analysis of the association between BCAA levels and incident CAD

According to the pre-specified search strategy, 6,431 articles were retrieved and seven articles including 38,975 participants were included in the final meta-analysis (Table S5). The retrieval process is shown in Figure S1. All literature scores were >7 points indicating a low risk of bias. The quality evaluation results are shown in Table S5.

The meta-analysis of seven studies was performed using a random-effects model (Figure S4). The RR was 1.18 (95%CI 1.11 to 1.24), indicating that BCAA was a risk factor for CAD. The heterogeneity was low (I^2 44.3%), and subgroup analysis suggested that the heterogeneity was mainly due to differences in regions and research methods (Figure S4).

Text S4. BCAA-associated SNPs with risk factors are not a suitable tool for MR analysis

The hypothesis of MR is that genetic variation as an instrumental variable is not pleiotropic. The existence of pleiotropic genetic variation is thus not suitable for MR. *SLC1A4* (rs17669826, rs2422358, and rs2007061) encodes a serine transporter related to the serine concentration[4]; *ABCG2* (rs116017006, rs7655059, rs62308058, and rs2911711) encodes a lipid metabolism-transferring protein[5]; *ASGR1* (rs7406661) is associated with decreased non-HDL cholesterol levels and a decreased risk of CAD[6]; rs6589564 and rs10750096 are located in the *BUN13-APOA5* region, which is known to be related to HDL and triglycerides[7]; rs2074216 and rs125651 are adjacent to *ELP5* and the expression of these two SNPs and ELP5 are related, while *ELP5* affects p53 and is an important factor in lipid-metabolism pathways[8]; *GCKR* (rs4665985, rs1260326, rs780105, rs1728918, and rs2911711) is a well-known pleiotropic gene associated with HDL, LDL, and triglycerides[9]; and *DDX19B* (rs12325419) regulates inositol hexakisphosphate,[10] leading to lipid abnormalities.[11] The above genetic variations affect confounding factors. We therefore excluded these genes to avoid the potential effects of the genes on confounding factors, which could mask the relationship between BCAA and CAD. We therefore only included BCAAs that were not affected by the pleiotropic effects of genetic mutations associated with confounding factors in the current MR analysis.

Table S1. Mendelian randomization estimates of the association between genetically predicted increases in serum leucine, isoleucine, and valine levels and coronary artery disease.

Amino acid	SNP	Beta	Se	OR (95%CI)	$p-$ Value
Leucine	11	0.076	0.036	1.08(1.01, 1.16)	0.036
Isoleucine	12	0.125	0.046	1.13(1.03, 1.24)	0.006
Valine	11	0.064	0.034	1.07(1.00, 1.14)	0.058

Table S2. Branched-chain amino acid-associated genetic variants screening data after applying a strict linkage disequilibrium cut-off (r^2<0.001 or lead SNP).

SNP	Nearby Gene	Chromosome	Effect/other Alleles	Effect Allele Frequncy	Beta(Se) of BCAAs Level per Alleles[1]	*p* − Value[1]	OR(95%CI) for CAD per Alleles[2]	*p* − Value[2]
**LD: r2<0.001**								
rs1420601	CBLN1	16	C/T	0.400	0.07(0.01)	3.63E−08	1.01(0.99, 1.03)	0.244
rs58101275	TRMT61A	14	G/A	0.790	0.09(0.02)	9.87E−10	1.01(0.99, 1.04)	0.222
rs9637599	PPM1K	4	C/A	0.470	0.11(0.01)	7.64E−36	1.00(0.98, 1.02)	0.757
rs1919128	C2orf16	2	G/A	0.275	0.07(0.01)	1.18E−10	1.01(0.99, 1.03)	0.422
**Lead SNP**								
rs1420601	CBLN1	16	C/T	0.400	0.07(0.01)	3.63E−08	1.01(0.99, 1.03)	0.244
rs58101275	TRMT61A	14	G/A	0.790	0.09(0.02)	9.87E−10	1.01(0.99, 1.04)	0.222
rs9637599	PPM1K	4	C/A	0.470	0.11(0.01)	7.64E−36	1.00(0.98, 1.02)	0.757
rs1919128	C2orf16	2	G/A	0.275	0.07(0.01)	1.18E−10	1.01(0.99, 1.03)	0.422
rs13030345	MRPL33	2	T/G	0.191	0.07(0.01)	4.52E−09	1.01(0.98, 1.03)	0.488

[1] Summary statistics per effect allele for serum branched-chain amino acid (BCAA) levels obtained from a genome-wide association meta-analysis.
[2] Effect size estimate (β coefficient, measured as log odds ratio of coronary artery disease [CAD] per additional BCAA-raising allele, and standard error) and P value for each single-nucleotide polymorphism in CAD were obtained from the CARDIoGRAMplusC4D consortium.

Table S3. Mendelian randomization effect size for branched-chain amino acid-associated single-nucleotide polymorphisms in cardiovascular disease after applying a strict linkage disequilibrium cut-off (r^2<0.001 or lead SNP).

Outcome	Beta	Se	p—Value
**LD: r^2<0.001 (4 SNP)**			
Coronary heart disease	0.089	0.058	0.126
Myocardial infarction	0.116	0.068	0.089
Heart attack/myocardial infarction	0.005	0.002	0.029
Stroke	0.111	0.058	0.056
Ischemic stroke	0.144	0.064	0.024
Ischemic stroke (cardioembolic)	0.371	0.140	0.008
Ischemic stroke (small-vessel)	0.235	0.144	0.103
Ischemic stroke (large artery atherosclerosis)	0.085	0.155	0.584
Subarachnoid haemmorrhage	-0.082	0.247	0.739
Intracerebral haemmorrhage	-0.069	0.220	0.752
Deep venous thrombosis	0.001	0.002	0.665
Pulmonary embolism	0.001	0.001	0.521
**Lead SNP (5 SNP)**			
Coronary heart disease	0.092	0.055	0.095
Myocardial infarction	0.117	0.061	0.056
Heart attack/myocardial infarction	0.006	0.002	0.010
Stroke	0.104	0.055	0.060
Ischemic stroke	0.137	0.061	0.024
Ischemic stroke (cardioembolic)	0.339	0.127	0.008
Ischemic stroke (small-vessel)	0.267	0.137	0.050
Ischemic stroke (large artery atherosclerosis)	0.098	0.148	0.506
Subarachnoid haemmorrhage	-0.065	0.235	0.782
Intracerebral haemmorrhage	0.092	0.298	0.758
Deep venous thrombosis	0.002	0.003	0.365
Pulmonary embolism	0.001	0.001	0.377

Table S4. Associations between genetically predicted serum branched-chain amino acid levels and cardiometabolic risk factors.

Outcome	Beta(se)	$p-$Value
LDL cholesterol	0.009(0.028)	0.750
HDL cholesterol	-0.069(0.021)	0.001
Triglycerides	0.222(0.142)	0.117
Total cholesterol	0.040(0.072)	0.579
HOMA-IR	-0.002(0.022)	0.919
Fasting glucose	-0.030 (0.029)	0.293
Fasting insulin	-0.002(0.017)	0.895
HbA1C	0.005(0.017)	0.772
2h glucose	-0.059(0.177)	0.739
BMI	0.047(0.36)	0.198
Waist-to-hip	0.015(0.020)	0.476
Cigarettes smoked per day	0.136(0.323)	0.674
Diastolic blood pressure	0.025(0.004)	0.00004
Systolic blood pressure	0.048(0.010)	2.75E-6
Type 2 diabetes	0.232(0.069)	0.0007

BMI, body mass index; CI, confidence interval; HOMA-IR, homeostatic model assessment of insulin resistance.

Table S5. Study characteristics

Study	Year	Country	Multi-centre	Number enrolled	Average age (SD) years	Quality Score
Tobias et al.**[12]**	2018	America	No	27041	54.4 (7.2)	9[&]
Ruiz et al.**[13]**	2016	Spain	No	970	67.2(6)	8[&]
Wen et al.**[14]**	2016	China	No	1302	59.05(5.98)	8[#]
R.Y. et al.**[15]**	2015	China	No	429	64.6(10.8)	8[&]
Bhattacharya et al.**[16]**	2014	America	No	1605	57(11.7)	8[&]
Wurtz et al.**[17]**	2014	Finland	No	7256	48(13)	8[&]
Shah et al.**[18]**	2010	America	No	348	60.4(5.1)	7[&]

[&] Newcastle-Ottawa Scale.

[#] Study was assessed using an 11-item checklist recommended by the Agency for Healthcare Research and Quality (AHRQ).

Figure S1. Workflow for systematic review of published observational studies examining the association between branched-chain amino acid levels and incident coronary artery disease.

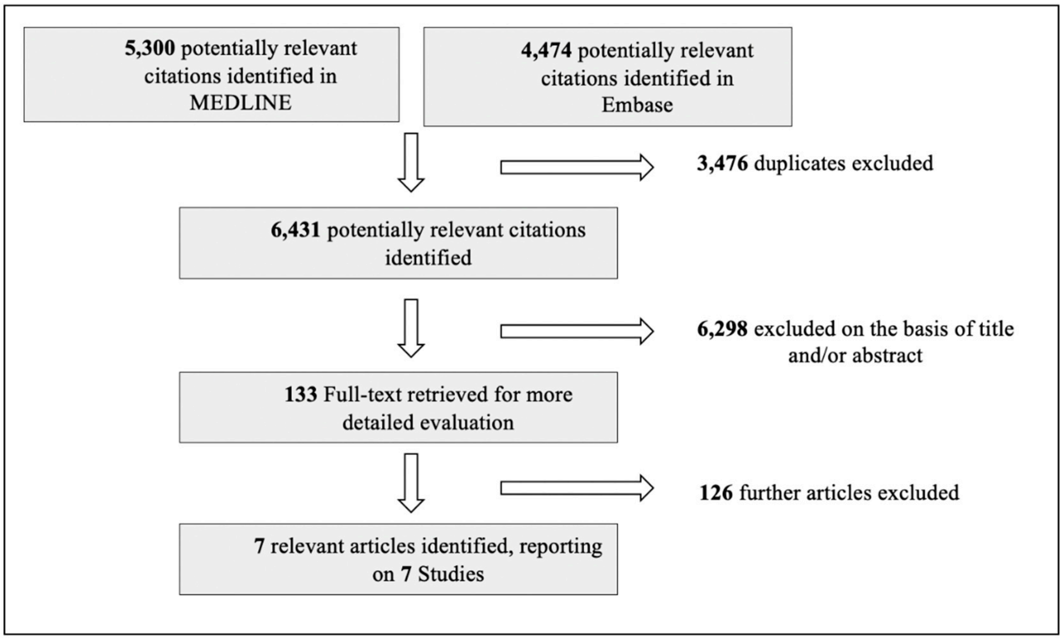

Figure S2. Scatter and leave-one-out plots demonstrating influential outliers in Mendelian randomization of branched-chain amino acids and coronary artery disease risk.

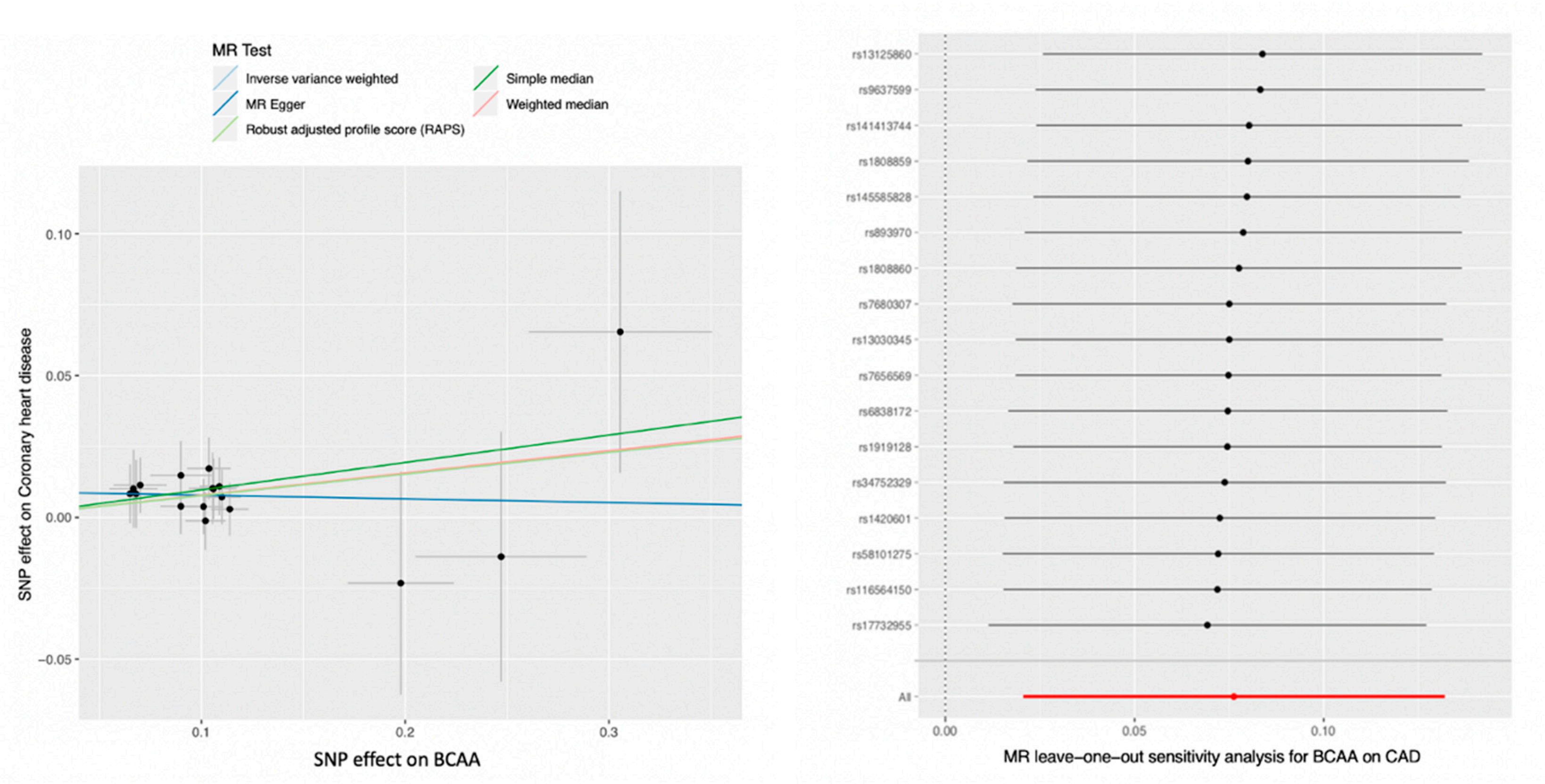

Figure S3. Mendelian randomization (MR) estimates of the association between genetically predicted increases in serum leucine, isoleucine, valine levels and coronary artery disease (CAD). (a) MR effect size for leucine-associated single-nucleotide polymorphisms (SNPs) on CAD. (b) MR effect size for isoleucine-associated SNPs on CAD. (c) MR effect size for valine-associated SNPs on CAD

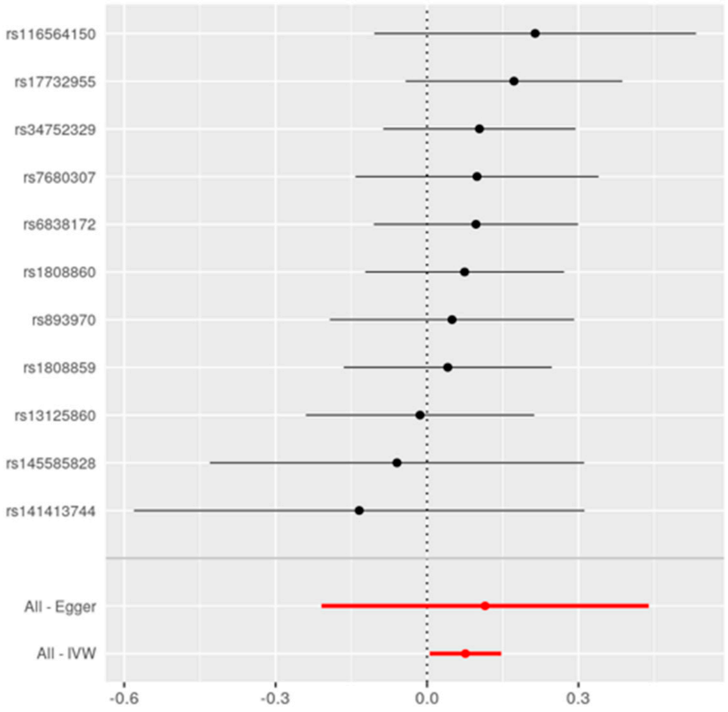

(a)

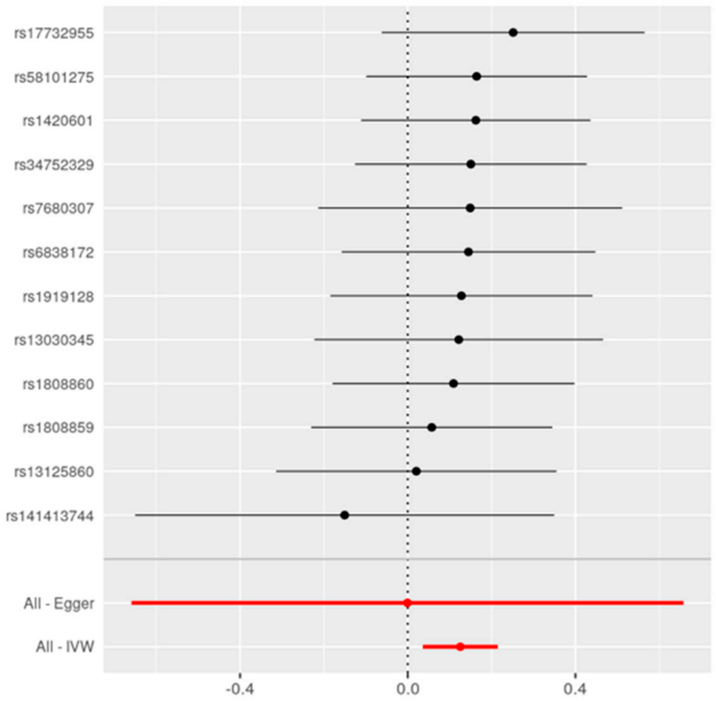

(b)

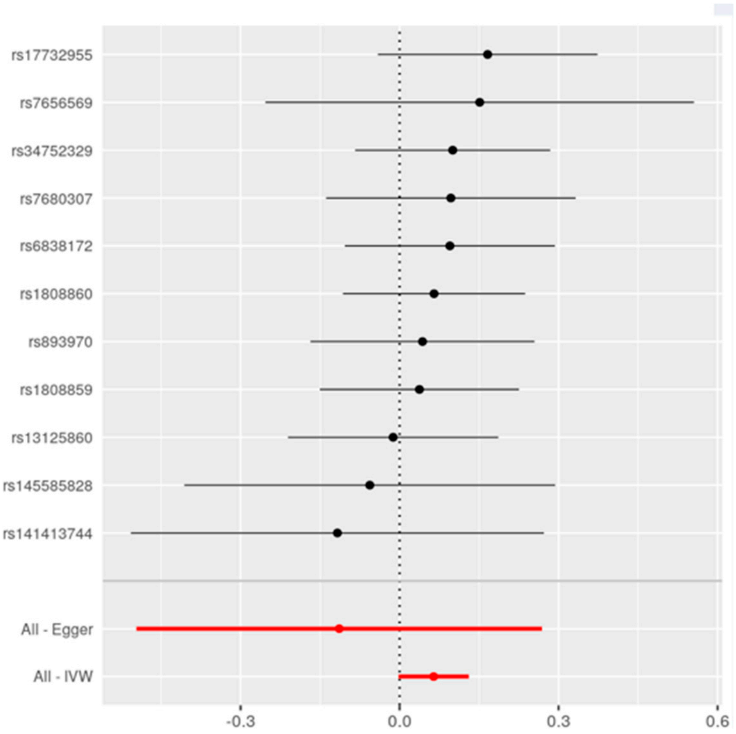
rs17732955
rs7656569
rs34752329
rs7680307
rs6838172
rs1808860
rs893970
rs1808859
rs13125860
rs145585828
rs141413744
All - Egger
All - IVW
-0.3
0.0
0.3
0.6

(c)

Figure S4. Meta-analysis of observational associations between baseline branched-chain amino acid levels and incident coronary artery disease. (a) All study subjects. (b) Different methods of study subjects. C-C, case-control studies.

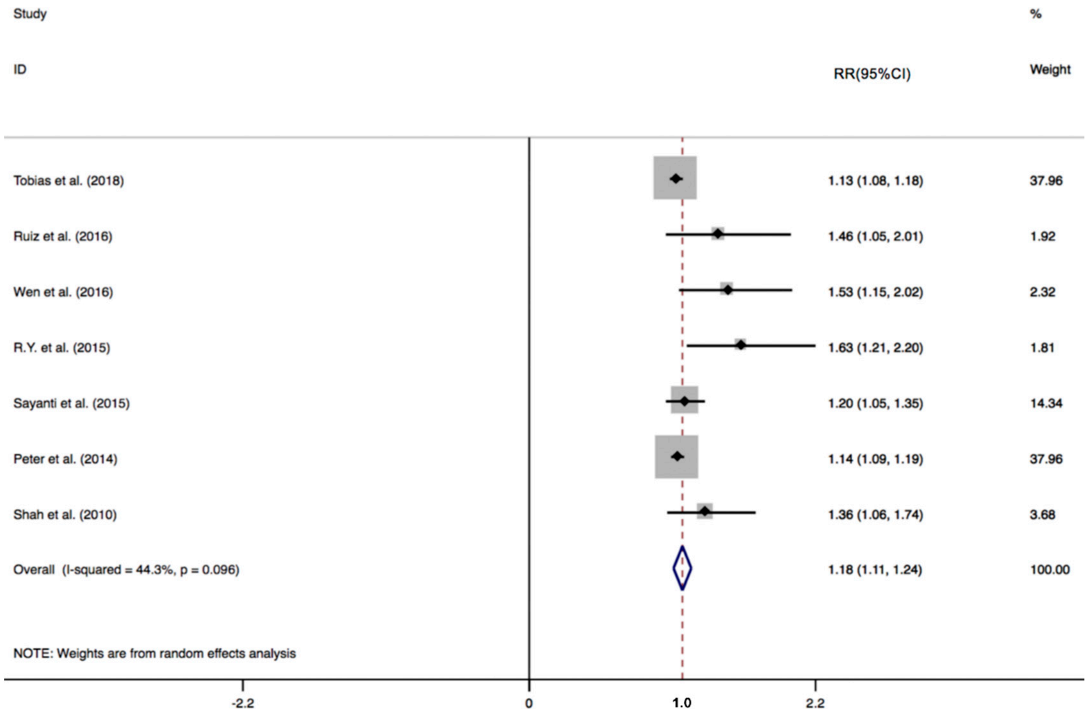

(a)

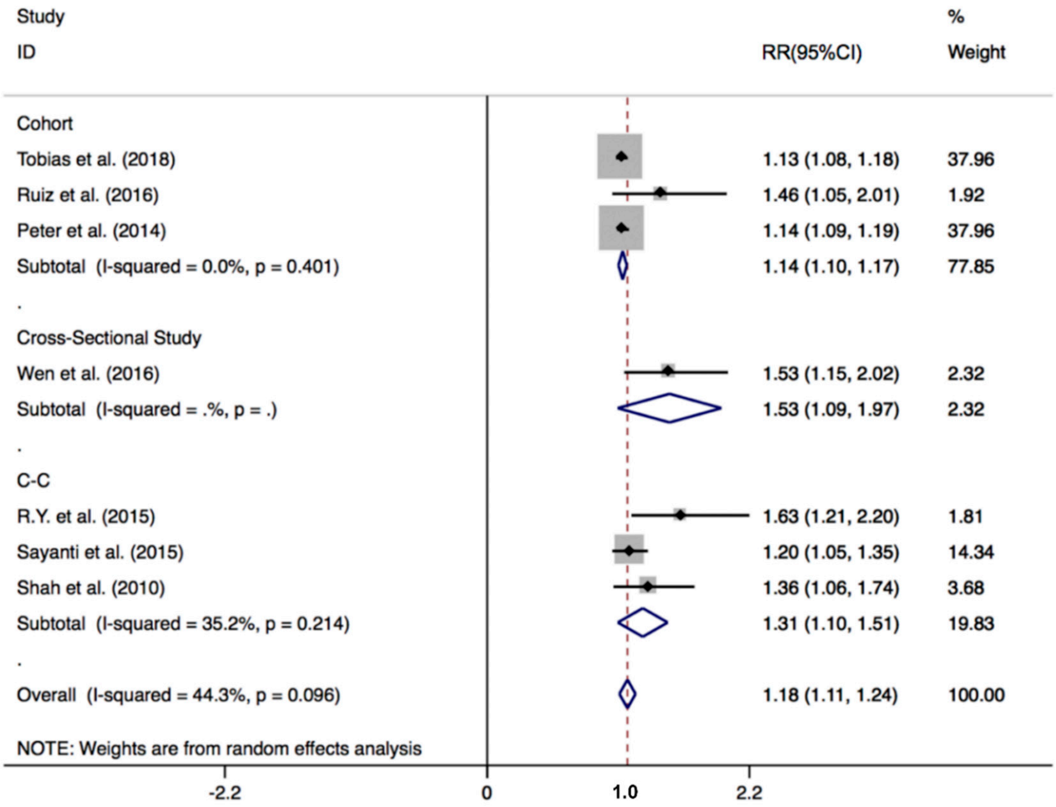
Study
ID
RR(95%CI)
%
Weight
Cohort
Tobias et al. (2018)
1.13 (1.08, 1.18)
37.96
Ruiz et al. (2016)
1.46 (1.05, 2.01)
1.92
Peter et al. (2014)
1.14 (1.09, 1.19)
37.96
Subtotal (I-squared = 0.0%, p = 0.401)
1.14 (1.10, 1.17)
77.85
Cross-Sectional Study
Wen et al. (2016)
1.53 (1.15, 2.02)
2.32
Subtotal (I-squared = .%, p = .)
1.53 (1.09, 1.97)
2.32
C-C
R.Y. et al. (2015)
1.63 (1.21, 2.20)
1.81
Sayanti et al. (2015)
1.20 (1.05, 1.35)
14.34
Shah et al. (2010)
1.36 (1.06, 1.74)
3.68
Subtotal (I-squared = 35.2%, p = 0.214)
1.31 (1.10, 1.51)
19.83
Overall (I-squared = 44.3%, p = 0.096)
1.18 (1.11, 1.24)
100.00
NOTE: Weights are from random effects analysis
-2.2
0
1.0
2.2

(b)

Figure S5. Gene expression enrichment of BCAA loci in GTEx tissues. (**a**) Tissue enrichment of PPM1K, TRMT61A, MRPL33, and CBLN1. (**b**)Gene expression enrichment of C2orf16 in GTEx tissues.

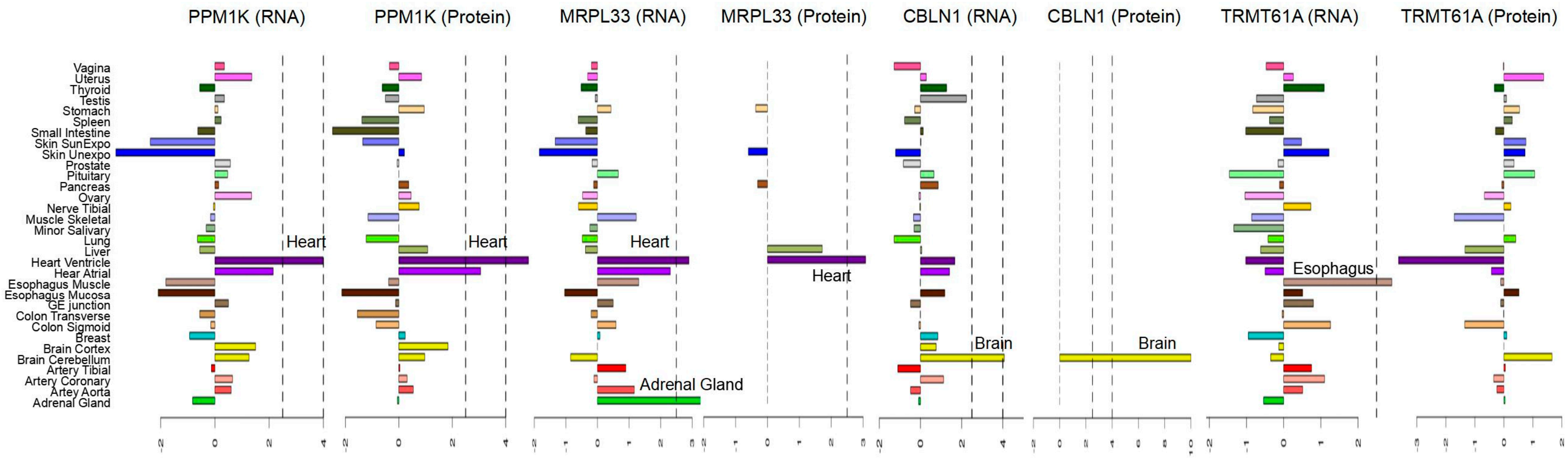

(a)

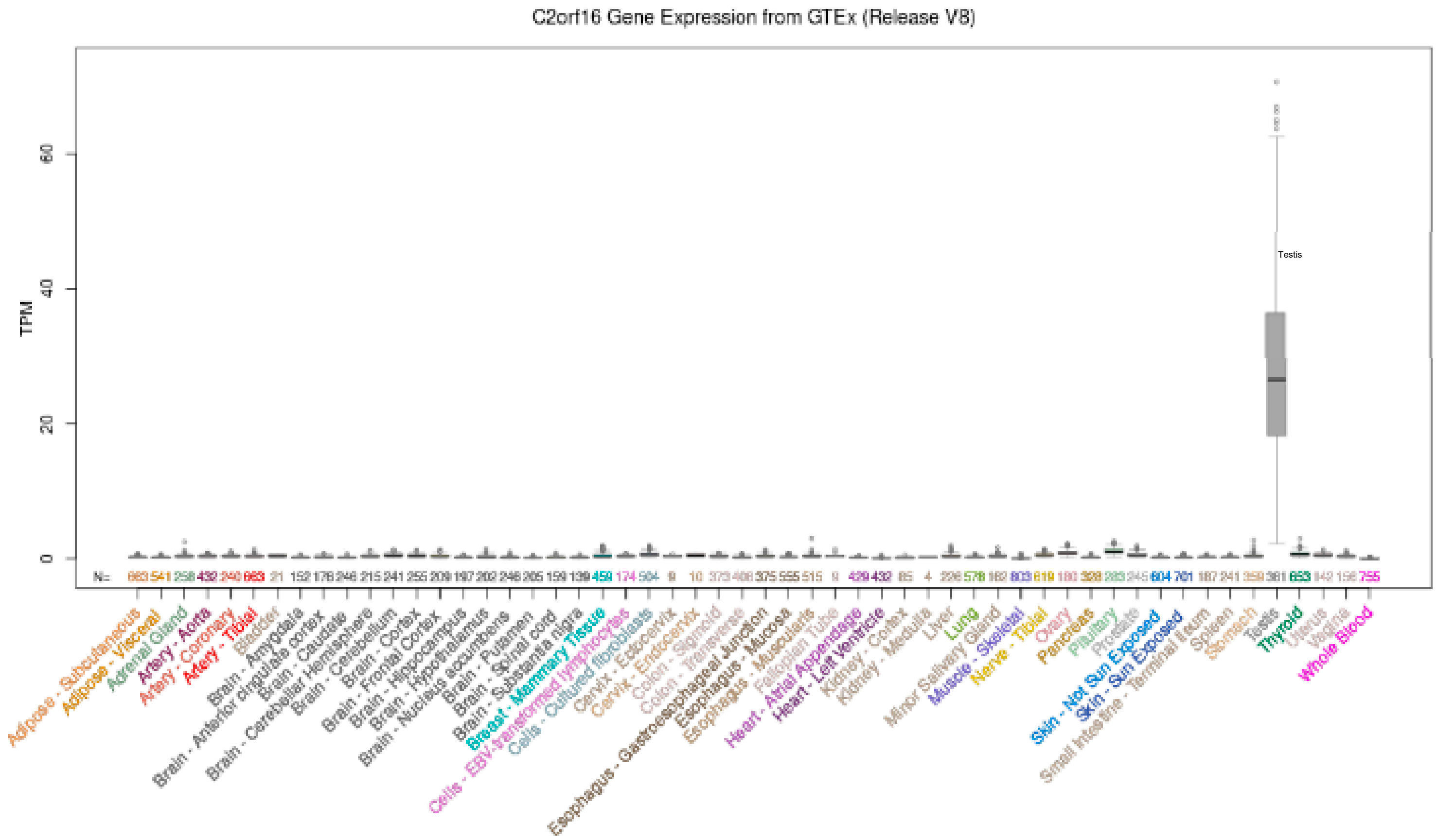
C2orf16 Gene Expression from GTEx (Release V8)
TPM
0
20
40
60
Testis
Adipose - Subcutaneous
Adipose - Visceral
Adrenal Gland
Artery - Aorta
Artery - Coronary
Artery - Tibial
Bladder
Brain - Amygdala
Brain - Anterior cingulate cortex
Brain - Caudate
Brain - Cerebellar Hemisphere
Brain - Cerebellum
Brain - Cortex
Brain - Frontal Cortex
Brain - Hippocampus
Brain - Hypothalamus
Brain - Nucleus accumbens
Brain - Putamen
Brain - Spinal cord
Brain - Substantia nigra
Breast - Mammary Tissue
Cells - EBV-transformed lymphocytes
Cells - Cultured fibroblasts
Cervix - Ectocervix
Cervix - Endocervix
Colon - Sigmoid
Colon - Transverse
Esophagus - Gastroesophageal Junction
Esophagus - Mucosa
Esophagus - Muscularis
Fallopian Tube
Heart - Atrial Appendage
Heart - Left Ventricle
Kidney - Cortex
Kidney - Medulla
Liver
Lung
Minor Salivary Gland
Muscle - Skeletal
Nerve - Tibial
Ovary
Pancreas
Pituitary
Prostate
Skin - Not Sun Exposed
Skin - Sun Exposed
Small Intestine - Terminal Ileum
Spleen
Stomach
Testis
Thyroid
Uterus
Vagina
Whole Blood

(b)

Figure S6. Tissue enrichment analysis based on gene expression. Tissue enrichment analysis was performed with MAGMA, using gene expression data for 30 tissues from GTEx and gene-based association statistics based on the BCAA genome-wide meta-analysis. The y-axis shows −log10 P values for enrichment for each tissue.

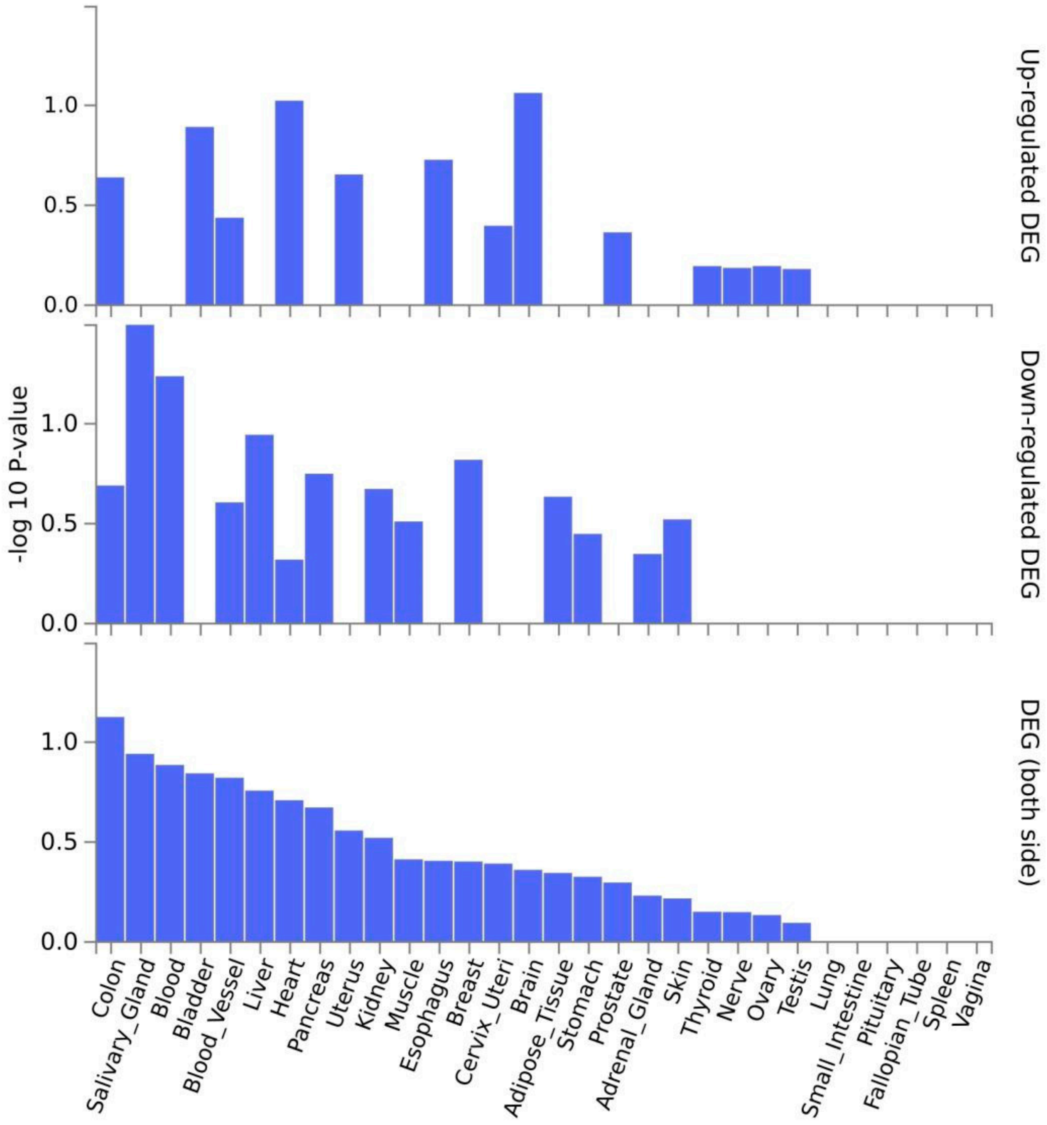

Figure S7. Summary of the catabolism pathways of BCAAs. The circulating pool of free BCAAs is determined by a balance between their input (e.g., diet, proteolysis) and output (e.g., protein synthesis, catabolism for energy). The first two steps in the pathway are common to all three BCAAs: the first reaction, catalysed by BCAT, is a reversible transamination to form BCKAs, and the second reaction, catalyzed by the BCKD complex, is an irreversible oxidative decarboxylation of BCKAs, and is the rate-limiting step in the overall BCAA catabolic pathway. BCAT, branched-chain aminotransferase. BCKAs, branched-chain α-keto acids. BCKD, branched-chain α-keto acid dehydrogenase.

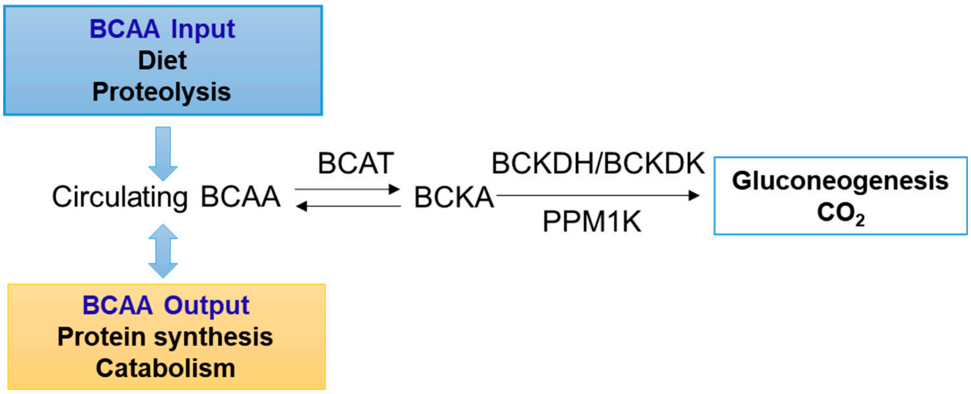

References

1. Xu, L.; Borges, M.C.; Hemani, G.; Lawlor, D.A. The role of glycaemic and lipid risk factors in mediating the effect of BMI on coronary heart disease: a two-step, two-sample Mendelian randomisation study. *Diabetologia* **2017**, *60*, 2210-2220, doi:10.1007/s00125-017-4396-y.
2. Richmond, R.C.; Hemani, G.; Tilling, K.; Davey Smith, G.; Relton, C.L. Challenges and novel approaches for investigating molecular mediation. *Human molecular genetics* **2016**, *25*, R149-R156, doi:10.1093/hmg/ddw197.
3. Hartwig, F.P.; Borges, M.C.; Horta, B.L.; Bowden, J.; Davey Smith, G. Inflammatory Biomarkers and Risk of Schizophrenia: A 2-Sample Mendelian Randomization Study. *JAMA Psychiatry* **2017**, *74*, 1226-1233, doi:10.1001/jamapsychiatry.2017.3191.
4. Arriza, J.L.; Kavanaugh, M.P.; Fairman, W.A.; Wu, Y.N.; Murdoch, G.H.; North, R.A.; Amara, S.G. Cloning and expression of a human neutral amino acid transporter with structural similarity to the glutamate transporter gene family. *Journal of Biological Chemistry* **1993**, *268*, 15329-15332.
5. Hegedus, C.; Telbisz, A.; Hegedus, T.; Sarkadi, B.; Ozvegy-Laczka, C. Lipid regulation of the ABCB1 and ABCG2 multidrug transporters. *Advances in cancer research* **2015**, *125*, 97-137, doi:10.1016/bs.acr.2014.10.004.
6. Nioi, P.; Sigurdsson, A.; Thorleifsson, G.; Helgason, H.; Agustsdottir, A.B.; Norddahl, G.L.; Helgadottir, A.; Magnusdottir, A.; Jonasdottir, A.; Gretarsdottir, S.; et al. Variant ASGR1 Associated with a Reduced Risk of Coronary Artery Disease. *N Engl J Med* **2016**, *374*, 2131-2141, doi:10.1056/NEJMoa1508419.
7. O'Brien, S.E.; Schrodi, S.J.; Ye, Z.; Brilliant, M.H.; Virani, S.S.; Brautbar, A. Differential Lipid Response to Statins Is Associated With Variants in the BUD13-APOA5 Gene Region. *Journal of cardiovascular pharmacology* **2015**, *66*, 183-188, doi:10.1097/FJC.0000000000000261.
8. Belalcazar, L.M.; Papandonatos, G.D.; McCaffery, J.M.; Peter, I.; Pajewski, N.M.; Erar, B.; Allred, N.D.; Balasubramanyam, A.; Bowden, D.W.; Brautbar, A.; et al. A common variant in the CLDN7/ELP5 locus predicts adiponectin change with lifestyle intervention and improved fitness in obese individuals with diabetes. *Physiological genomics* **2015**, *47*, 215-224, doi:10.1152/physiolgenomics.00109.2014.
9. Locke, A.E.; Kahali, B.; Berndt, S.I.; Justice, A.E.; Pers, T.H.; Day, F.R.; Powell, C.; Vedantam, S.; Buchkovich, M.L.; Yang, J.; et al. Genetic studies of body mass index yield new insights for obesity biology. *Nature* **2015**, *518*, 197-206, doi:10.1038/nature14177.
10. Okamura, M.; Yamanaka, Y.; Shigemoto, M.; Kitadani, Y.; Kobayashi, Y.; Kambe, T.; Nagao, M.; Kobayashi, I.; Okumura, K.; Masuda, S. Depletion of mRNA export regulator DBP5/DDX19, GLE1 or IPPK that is a key enzyme for the production of IP6, resulting in differentially altered cytoplasmic mRNA expression and specific cell defect. *PloS one* **2018**, *13*, e0197165, doi:10.1371/journal.pone.0197165.
11. Singh, A.; Bhatnagar, N.; Pandey, A.; Pandey, G.K. Plant phospholipase C family: Regulation and functional role in lipid signaling. *Cell Calcium* **2015**, *58*, 139-146, doi:10.1016/j.ceca.2015.04.003.

12. Tobias, D.K.; Lawler, P.R.; Harada, P.H.; Demler, O.V.; Ridker, P.M.; Manson, J.E.; Cheng, S.; Mora, S. Circulating Branched-Chain Amino Acids and Incident Cardiovascular Disease in a Prospective Cohort of US Women. *Circulation Genomic & Precision Medicine* **2018**, *11*, e002157.
13. Ruiz-Canela, M.; Toledo, E.; Clish, C.B.; Hruby, A.; Liang, L.; Salas-Salvado, J.; Razquin, C.; Corella, D.; Estruch, R.; Ros, E.; et al. Plasma Branched-Chain Amino Acids and Incident Cardiovascular Disease in the PREDIMED Trial. *Clinical chemistry* **2016**, *62*, 582-592, doi:10.1373/clinchem.2015.251710.
14. Hu, W.; Sun, L.; Gong, Y.; Zhou, Y.; Yang, P.; Ye, Z.; Fu, J.; Huang, A.; Fu, Z.; Yu, W.; et al. Relationship between Branched-Chain Amino Acids, Metabolic Syndrome, and Cardiovascular Risk Profile in a Chinese Population: A Cross-Sectional Study. *Int J Endocrinol* **2016**, *2016*, 8173905, doi:10.1155/2016/8173905.
15. Yang, R.Y.; Wang, S.M.; Sun, L.; Liu, J.M.; Li, H.X.; Sui, X.F.; Wang, M.; Xiu, H.L.; Wang, S.; He, Q.; et al. Association of branched-chain amino acids with coronary artery disease: A matched-pair case-control study. *Nutr Metab Cardiovasc Dis* **2015**, *25*, 937-942, doi:10.1016/j.numecd.2015.06.003.
16. Bhattacharya, S.; Granger, C.B.; Craig, D.; Haynes, C.; Bain, J.; Stevens, R.D.; Hauser, E.R.; Newgard, C.B.; Kraus, W.E.; Newby, L.K.; et al. Validation of the association between a branched chain amino acid metabolite profile and extremes of coronary artery disease in patients referred for cardiac catheterization. *Atherosclerosis* **2014**, *232*, 191-196, doi:10.1016/j.atherosclerosis.2013.10.036.
17. Wurtz, P.; Havulinna, A.S.; Soininen, P.; Tynkkynen, T.; Prieto-Merino, D.; Tillin, T.; Ghorbani, A.; Artati, A.; Wang, Q.; Tiainen, M.; et al. Metabolite profiling and cardiovascular event risk: a prospective study of 3 population-based cohorts. *Circulation* **2015**, *131*, 774-785, doi:10.1161/CIRCULATIONAHA.114.013116.
18. Shah, S.H.; Bain, J.R.; Muehlbauer, M.J.; Stevens, R.D.; Crosslin, D.R.; Haynes, C.; Dungan, J.; Newby, L.K.; Hauser, E.R.; Ginsburg, G.S.; et al. Association of a peripheral blood metabolic profile with coronary artery disease and risk of subsequent cardiovascular events. *Circulation. Cardiovascular genetics* **2010**, *3*, 207-214, doi:10.1161/CIRCGENETICS.109.852814.
